# A model of audio–visual motion integration during active self-movement

**DOI:** 10.1167/jov.25.2.8

**Published:** 2025-02-19

**Authors:** Maria Gallagher, Joshua D. Haynes, John F. Culling, Tom C. A. Freeman

**Affiliations:** 1 School of Psychology, University of Kent, Canterbury, UK; 2 School of Psychology, Cardiff University, Cardiff, UK; 3 School of Health Sciences, University of Manchester, Manchester, UK

**Keywords:** multisensory integration, motion perception, self-movement, active movement, audio–visual integration

## Abstract

Despite good evidence for optimal audio–visual integration in stationary observers, few studies have considered the impact of self-movement on this process. When the head and/or eyes move, the integration of vision and hearing is complicated, as the sensory measurements begin in different coordinate frames. To successfully integrate these signals, they must first be transformed into the same coordinate frame. We propose that audio and visual motion cues are separately transformed using self-movement signals, before being integrated as body-centered cues to audio–visual motion. We tested this hypothesis using a psychophysical audio–visual integration task in which participants made left/right judgments of audio, visual, or audio–visual targets during self-generated yaw head rotations. Estimates of precision and bias from the audio and visual conditions were used to predict performance in the audio–visual conditions. We found that audio–visual performance was predicted well by models that suggested the transformation of cues into common coordinates but could not be explained by a model that did not rely on coordinate transformation before integration. We also found that precision specifically was better predicted by a model that accounted for shared noise arising from signals encoding head movement. Taken together, our findings suggest that motion perception in active observers is based on the integration of partially correlated body-centered signals.

## Introduction

Despite good evidence for optimal audio–visual integration of spatial cues to direction and movement in stationary observers, few studies have considered the impact of self-movement on this process. Early auditory and visual signals are represented in different spatial coordinate frames, with auditory cues starting out in head-centered coordinates and visual signals in eye-centered coordinates. The coordinate frames align in observers who keep their eyes and head stationary, which makes the integration of audio–visual spatial cues relatively straightforward. Observers appear to use an optimal integration strategy, based on the maximum likelihood principles original developed to explain the integration of depth cues both within and across modalities (Ernst & Banks, 2002; Ernst & Bülthoff, 2004; Landy, Maloney, Johnston, & Young, 1995). Accordingly, more reliable estimates are given a higher weighting than less reliable ones, resulting in a combined percept that is more precise than either cue alone. The maximum-likelihood principle explains why the spatial localization of stationary audio–visual targets is dominated by the visual location when visual reliability is high, but gradually shifts toward the auditory location as visual reliability decreases (Alais & Burr, 2004b; Bolognini, Leor, Passamonti, Stein, & Làdavas, 2007). It also explains why localization is more precise for audio–visual targets presented in isolation compared with auditory or visual stimuli alone (Alais & Burr, 2004b; Hairston, Laurienti, Mishra, Burdette, & Wallace, 2003; Wuerger, Meyer, Hofbauer, Zetzsche, & Schill, 2010). The optimal integration exhibited by observers is not limited to stationary targets, with the perceived direction of moving auditory and visual targets shifting toward the direction of the more reliable sense (Meyer & Wuerger, 2001; Soto-Faraco, Lyons, Gazzaniga, Spence, & Kingstone, 2002; Soto-Faraco, Spence, Lloyd, & Kingstone, 2004).

The integration of audio–visual cues is made more complicated when the observer moves their head and/or eyes because self-movement impacts vision and hearing in different ways. For example, with the head stationary, moving the eyes across a stationary audio–visual object results in motion across the retina, but none across the ears. In this case, the coordinate frames in which the visual and auditory information reside no longer coincide. As such, additional computational steps are required, first to “compensate” for movement of the eyes, and second to transform both modalities into a common coordinate frame. Only then is integration possible (Andersen, Snyder, Li, & Stricanne, 1993; Burns & Blohm, 2010; Harris, 1994; Landy et al., 1995; Sober & Sabes, 2003).

Previous research suggests that both auditory and visual signals can be transformed into eye-, head-, and/or body-centered coordinate frames, depending on the task and stimuli used (Cohen & Andersen, 2002; Collins, Heed, & Röder, 2010; Furman & Gur, 2012; Hairston et al., 2003; Kopinska & Harris, 2003; Wallach, 1987; Wertheim, 1994). During smooth pursuit eye movements, information from eye-muscle proprioception and efference copy is integrated with retinal information to distinguish retinal motion resulting from self-movement and object movement (Freeman & Banks, 1998; Furman & Gur, 2012; Sperry, 1950; von Holst & Mittelstaedt, 1950). It is likely that the compensation for eye movements occurs in the medial–superior temporal (MST) cortical region, which receives extraretinal input from proprioception, the vestibular system, and efference copy and represents visual signals in both eye- and head-centered reference frames (Furman & Gur, 2012; Gu, Angelaki, & DeAngelis, 2008; Newsome, Wurtz, & Komatsu, 1988; Ono & Mustari, 2012). Less attention has been given to how the auditory system compensates for head movements, despite evidence that individuals are able to localize auditory stimuli during head and eye movements (Genzel, Firzlaff, Wiegrebe, & MacNeilage, 2016; Goossens & van Opstal, 1999). It is possible that head-movement compensation occurs via processes similar to those for eye-movement compensation, requiring the integration of auditory spatial information with “extracochlear” signals from proprioception, the vestibular system, and efference copy (Freeman, Culling, Akeroyd, & Brimijoin, 2017). Previous research has demonstrated that such signals can influence the localization of auditory cues (Lewald, Karnath, & Ehrenstein, 1999; Lewald & Karnath, 2000), with evidence suggesting that auditory signals are transformed from head- to body-centered coordinates (Genzel et al., 2016; Goossens & van Opstal, 1999; Vliegen, Van Grootel, & Van Opstal, 2004).

**Figure 1.**
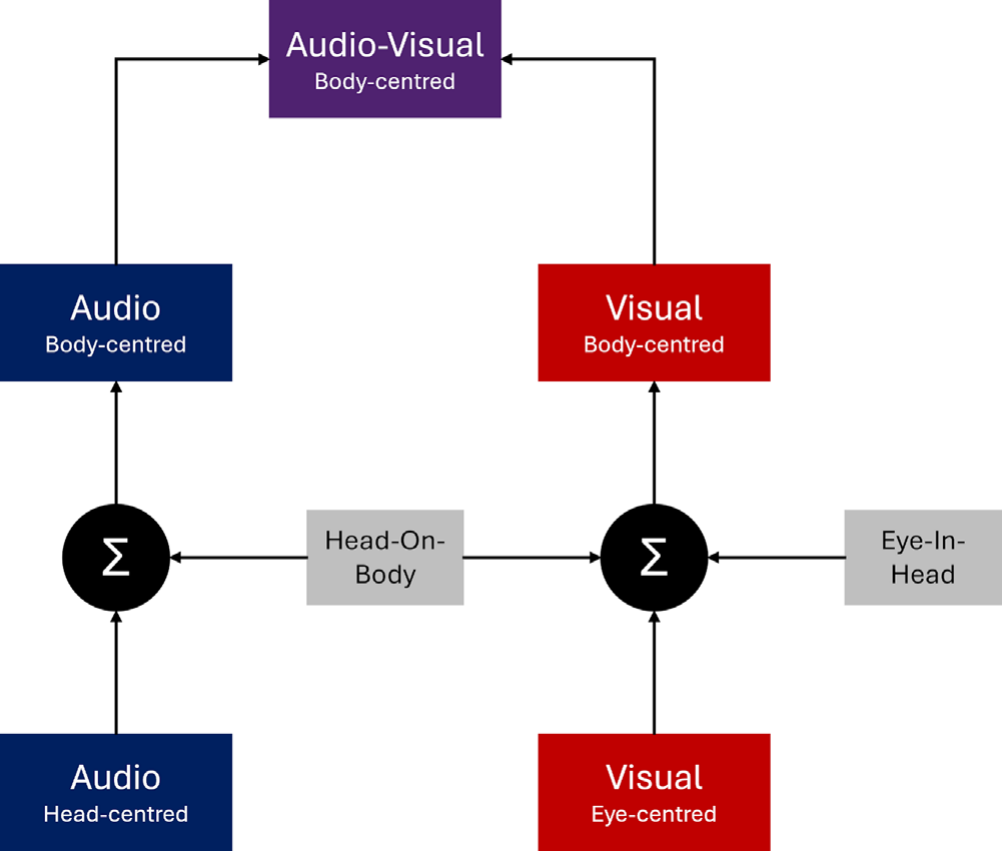
Outline of the proposed framework for integrating audio–visual cues during self-movement. The framework is based on the integration of compensated cues. For hearing, compensation is carried out using head-on-body signals to transform audio-motion signals from head-centered coordinates to body-centered coordinates. For vision, both head-on-body and additional eye-in-head signals are used to transform visual motion signals from eye-centered coordinates to body-centered coordinates. These compensated audio and visual signals are then integrated, resulting in a body-centered audio–visual estimate of motion.

Our hypothesis is that audio–visual motion integration during self-movement is based on the optimal combination of compensated auditory and visual cues, as shown in Figure 1. According to this hypothesis, auditory and visual cues are first transformed into a common coordinate frame, based on self-movement compensation mechanisms described above, and then are integrated into a coherent audio–visual percept using optimal integration of the compensated cues. We make the assumption that the cues are transformed into a body-centered reference frame partly because this reference frame remains stationary during head and eye movements in our experiments, and also previous literature shows that both auditory and visual cues are transformed into this frame (Furman & Gur, 2012; Goossens & van Opstal, 1999; Kopinska & Harris, 2003; Lewald et al., 1999). For example, Kopinska and Harris (2003) demonstrated that both auditory localization and visual localization were impacted by head-on-body position but were not affected by eye-in-head or body-in-space positions, implying that such localization judgments are based on body-centered coordinates. This is echoed in gaze-orienting behavior toward sequences of auditory and visual targets (Goossens & van Opstal, 1999). Crucially, as Figure 1 shows, the transformed cues are partially correlated because they share a source of noise that depends on the precision of the signals encoding head movement (the body-centered cues may be also biased, a point taken up below). To account for this shared noise, we used a modified version of the standard optimal cue combination model proposed by Oruç, Maloney, and Landy (2003). Instead of including the shared noise as a free parameter, however, we used a recently developed technique for measuring the precision of this self-movement signal, one specifically designed to measure signal noise when self-movement is self-controlled as in the current paper (Haynes, Gallagher, Culling, & Freeman, 2024).

We tested the framework sketched in Figure 1 using a psychophysical audio–visual integration task in which participants freely rotated their head around a vertical axis while maintaining their gaze on a head-stationary fixation point. Although an unusual fixation strategy, this type of gaze behavior guarantees that vision must transform retinal image motion into body-centered coordinates in order to make the correct spatial judgment, in the same way that hearing must transform auditory images. Participants were asked to judge the direction of motion of audio, visual, and audio–visual targets in interleaved conditions. In some conditions, we also added external noise to the stimuli to manipulate the reliability (i.e., precision) of the cues directly (Bentvelzen, Leung, & Alais, 2009; Ernst & Banks, 2002; Girshick, Landy, & Simoncelli, 2011). We fit the data with three models: a standard cue integration model (Ernst & Banks, 2002; Ernst & Bülthoff, 2004) based on transformed auditory and visual signals but which does not account for shared noise between the cues (body-centered integration); a modified optimal integration model (Oruç et al., 2003), based on transformed auditory and visual signals, which also accounts for shared noise (body-centered integration, adjusted for shared noise); and, finally, an optimal integration model based on uncompensated cues, i.e., auditory and visual image motion alone (image-centered integration). In anticipation of the results, we found that the data were accounted for well by models based on transformed auditory and visual signals but could not be explained by a model based on uncompensated cues (i.e., auditory and visual image motion alone).

## Methods

### Participants

Six participants (three female; mean age, 36.83 ± 14.74 years) completed the experiment. Two participants were naïve to the purposes of the study, and four participants were the study authors. No participants reported neurological or psychiatric conditions. All participants were right handed.

### Ethics

The study procedure was approved by the Cardiff University ethics committee (EC.12.04.03.3123GRA2), and the study was conducted in accordance with the tenets of the Declaration of Helsinki. All participants provided informed consent prior to commencing the study.

### Stimuli and equipment

**Figure 2.**
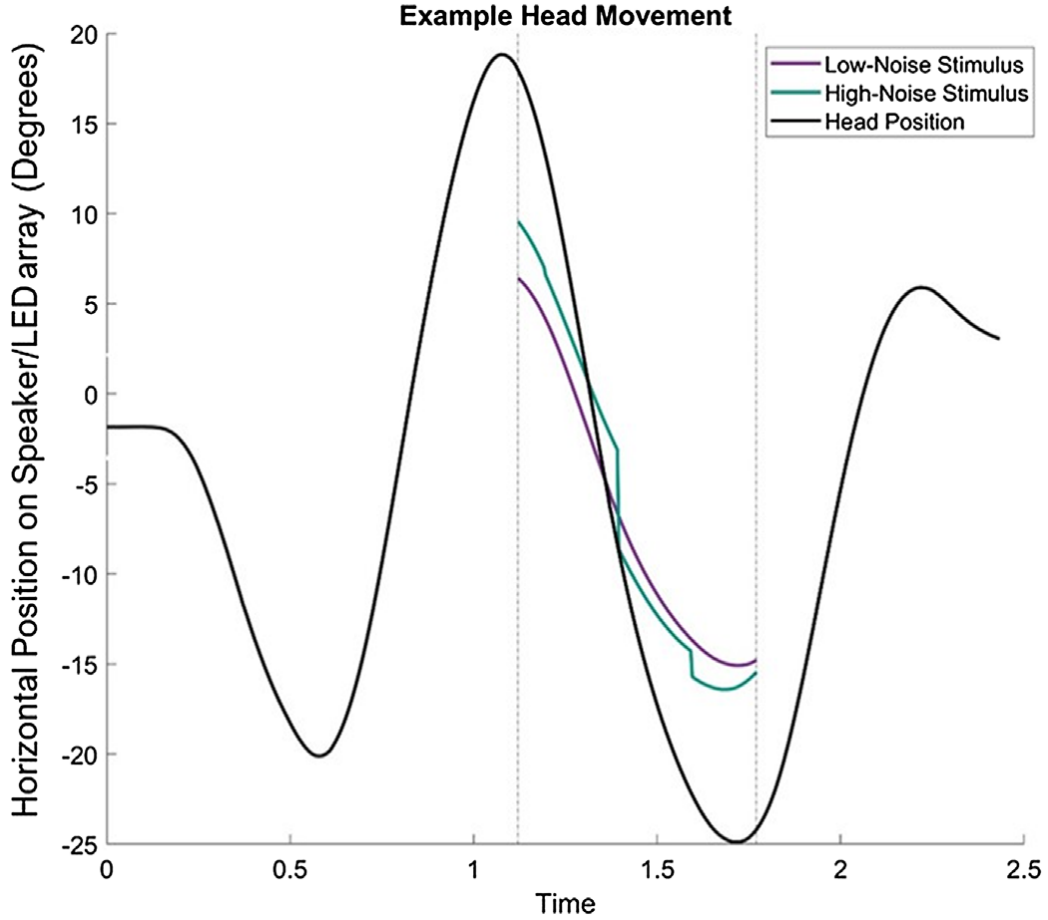
Example head movement and stimulus position over time. Vertical lines indicate the start and end of the third head sweep, during which time the stimulus to be judged was visible. The purple line indicates a low noise stimulus, and the green line indicates a high noise stimulus (i.e., jittered).

A photograph of the experimental setup can be seen Haynes et al. (2024). The experiment took place in a carpeted, sound-treated room with wall and ceiling tiles with absorption coefficients of 0.9, resulting in a reverberation time of ∼60 ms. Auditory stimuli were presented via a 2.4-meter-diameter ring of 48 speakers (Cambridge Audio Minx, London, UK), controlled by two 24-channel sound cards (MOTU, Cambridge, MA), each linked to four six-channel amplifiers (Auna, London, UK). Sound intensity was normalized across all speakers with a RadioShack 33-2055 digital meter on the “dB A” setting using 0.2- to 20-kHz noise. Auditory stimuli were spatially updated at 240 Hz. Visual stimuli were presented via a NeoPixel strip of 342 RGB LEDs subtending 256° (Adafruit Industries, New York, NY), controlled by a Uno microcontroller (Arduino, Monza, Italy). The LED strip was covered with a 1.2*f* neutral-density filter and three layers of diffuser gel at a distance of 35 mm. Visual stimuli were updated at 40 Hz. The distance from the participant's head to the LED/speaker array was 1.2 meters. Head movements were recorded via a LIBERTY tracker (Polhemus, Burlington, VT), sampled at 240 Hz. Changes in head-movement direction were detected using a smoothed derivative of head position, which was achieved by convolving tracker samples with a finite-difference filter (13 samples long). This meant that head turns were detected 7 frames (∼30 ms) after the turn was made (Figure 2). Eye movements were recorded via a Pupil Core eye tracker (Pupil Labs, Berlin, Germany) sampled at 30 Hz (participants 1 and 2, and first four sessions of participant 4) or 120 Hz (participants 5 and 6, and remainder of participant 4). The front-facing camera was used to calibrate eye position using a 3 × 2 array of calibration points, allowing conversion of normalized units to degrees. Stimulus presentation and response collection were conducted via custom MATLAB r2018b scripts (MathWorks, Natick, MA).

A visual cue was used to indicate the beginning of each trial. The cue was a diffuse blue LED blob, spatially windowed by a Gaussian distribution with σ = 1.05°. This light lasted for 500 ms or until the participant moved their head (whichever was sooner).

The fixation point was a diffuse green LED blob windowed by a Gaussian with σ = 1.05°, increased to σ = 1.07° by the diffuser, with a peak luminance of ∼0.042 cd/m^2^. The fixation point was yoked to the participant’s head movement, such that the eyes were always straight forward in the head (i.e., the eyes moved with respect to the speaker/LED ring).

The visual stimulus to be judged was a diffuse red LED blob windowed by a Gaussian with σ = 2.25° The auditory stimulus was a white noise burst spatially windowed by a Gaussian function with a standard deviation (*SD*) of 5.25° in power (σ = 7.5° amplitude). The noise was sampled at 48 kHz, with a peak of 70 dB. In audio–visual conditions, auditory and visual stimuli were presented at the same location with the same speed.

The method of constant stimuli was used to measure precision and accuracy. Audio and visual stimuli moved at a proportion of the head speed (movement gain), in the same or opposite direction as the head. Movement gains ranged from 0 to ± 0.5 of the proportion of head speed in seven steps (13 speeds total). Each speed was presented 30 times. Head movement speeds were entirely paced by the participants. However, no fixation or stimuli would appear if the participant’s head speed fell below a threshold (15°/s), as determined by a leaky integrator. This integrator made the amplitude/luminance of the stimulus decrease by 50% for each frame spent below the threshold speed. The precision of the stimuli could be modified by adding positional jitter (Bentvelzen et al., 2009) randomly drawn from a rectangular distribution that was ±7.5° wide. The jitter was updated at 5 Hz and was added to each modality to create low-noise and high-noise conditions (Figure 2; Table 1).

**Table 1.** Conditions used in the experiment.

Modality	Positional jitter
Visual	None
Visual
Audio	None
Audio
Audio–visual	None
Visual
Audio
Audio and visual

### Procedure

Each trial began with a cue light to indicate that the participants should position their head looking straight ahead and aligned with the body. When the participant was in the correct position, the cue light disappeared, and the participant was instructed to begin moving their head. Participants made self-paced back-and-forth (yaw) head movements while maintaining fixation on the green fixation light. On the third head sweep, a stimulus to be judged was presented, which moved in the same or opposite direction as the head, at a speed defined by the movement gain selected for that trial. The stimulus could be audio, visual, or audio–visual and could be presented at high or low reliabilities (with low reliability based on the addition of positional jitter). Thus, all of the conditions defined by Table 1 were interleaved in any one session. The participant judged whether the stimulus moved to the left or right using a key press. When the press was recorded, the next trial began once the participant's head was approximately centered (within ±7.5° of straight ahead). Eight conditions were tested (Table 1), with all trial types interleaved. In total, the experiment consisted of 3120 trials per participant (8 conditions × 13 target speeds × 30 target repetitions), split into approximately 24 10-minute blocks over six to eight sessions on separate days.

### Psychophysical analysis

**Figure 3.**
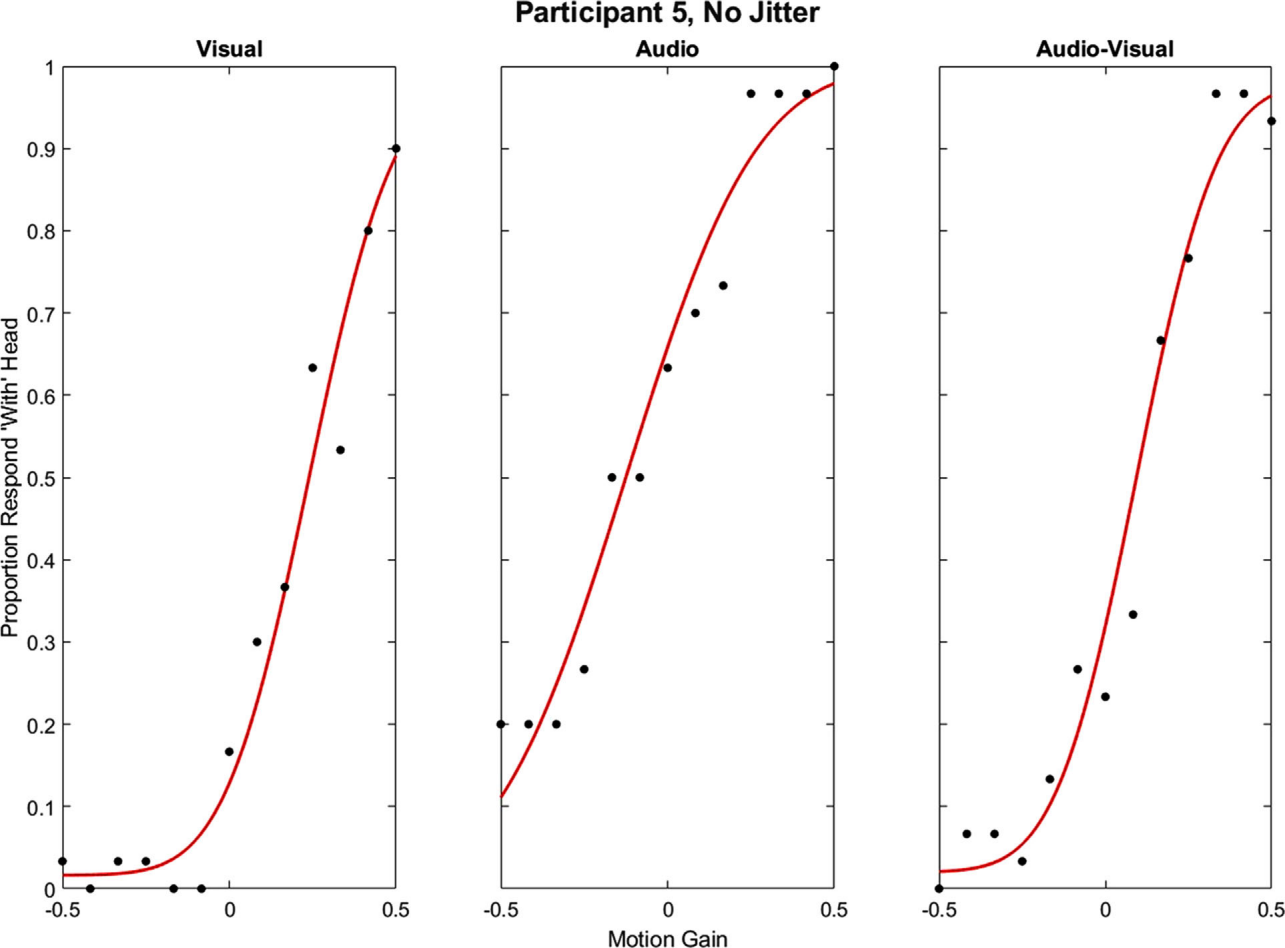
Example psychometric function fit from a naïve participant.

Analyses were carried out using MATLAB 9.12.0.1884302 (R2022a), Palamedes Toolbox (psychometric function fits, model estimations, and confidence intervals) (Prins & Kingdom, 2018), R 4.3.2 (R Core Team, 2022), and RStudio 2023.12.0 (RStudio Team, 2019), with packages ez (Lawrence, 2016) and dplyr (Wickham, François, Henry, Müller, & Vaughan, 2023) for repeated-measures ANOVAs and post hoc tests. The proportion of “with the head” responses was calculated for each gain value in each condition. Psychometric functions based on cumulative Gaussians were fit to the data using the PAL_PFML_Fit function from the Palamedes Toolbox (Prins & Kingdom, 2018). Biases were estimated at the point of 50% “with the head” responses. Precision was estimated as the inverse of the slope of the psychometric function. This corresponds to the standard deviation of cumulative Gaussian fit to the data. Accordingly, higher standard deviations indicate lower precision. Lapse rates were a free parameter, constrained to a maximum of 0.02 (Prins, 2012). Example psychometric function fits from a naïve subject in the visual, audio, and audio–visual no-jitter conditions can be seen in Figure 3.

### Models

Data in each of the four audio–visual conditions (no jitter, visual jitter, audio jitter, audio–visual jitter) were fit with three alternative models based on parameters obtained in the audio and visual conditions (see Table 2 for how the unimodal prediction conditions were coupled to appropriate audio–visual conditions). Model 1 was based on optimal integration of body-centered cues but ignoring the shared noise defined by the self-movement signal common to both compensated cues. Model 2 included this shared noise, and Model 3 was based on more standard optimal integration from auditory and visual image motion alone (i.e., self-movement signals were ignored). The parameters for these models were fixed, including the shared noise in Model 2, which was measured in a separate experiment on the same participants (Supplementary Appendix A). The only free parameters were those relating to the initial fitting of the psychometric functions to the raw data to obtain relevant points of subjective equality (PSEs) and slope parameters required to calculate the model predictions. Hence, there was no need to correct for the number of free parameters when comparing models because each had the same. To evaluate which model best predicted precision and bias on a group level, we calculated the squared differences of the model predictions to the observed values. We then compared these squared differences using repeated-measures ANOVAs, with Model and Condition as factors. Significant main effects and/or interactions were followed up with post hoc Bonferroni-corrected pairwise *t*-tests where necessary. We also compared the difference between each prediction and measured parameter on an individual level using 95% confidence intervals (CIs) constructed from 2000 bootstrapped samples. The CIs that did not contain 0 indicated that the prediction significantly differed from the actual parameter on an individual basis. Moreover, in a second analysis based on the same data using techniques in Haynes et al. (2024), we also accounted for the head-movement variability, which potentially affects the interpretation of the slopes of the psychometric functions in ways described below. Importantly, this second analysis tested the models at the level of the psychometric function, rather than on the basis of psychometric function parameters from the single-cue conditions. The root mean square error (RMSE) of each model fit to the data was calculated, and the differences in RMSE across Model and Condition were analyzed using repeated-measures ANOVAs as above. Thus, this second analysis provided further converging evidence to support our conclusions.

**Table 2.** Audio–visual conditions by predicting conditions.

Audio–visual condition	Predicting conditions
	Visual: no positional jitter
No jitter	
	Audio: no positional jitter
	Visual: positional jitter
Visual jitter	
	Audio: no positional jitter
	Visual: no positional jitter
Audio jitter	
	Audio: positional jitter
	Visual: positional jitter
Audio–visual jitter	
	Audio: positional jitter

#### Model 1: Body-centered integration

In the body-centered integration (BCI) model, we used a standard cue integration approach (Ernst & Banks, 2002; Ernst & Bülthoff, 2004) to predict the audio–visual bias (${\hat{S}}_{av}$) based on the weighted sum of the compensated audio (${\hat{S}}_{a}$) and visual biases (${\hat{S}}_{v}$):

(1) $$\begin{array}{c} {\hat{S}}_{av}=\;w_{a}{\hat{s}}_{a}+w_{v}{\hat{s}}_{v}\; \end{array}$$

Bias was defined as the PSE of the cumulative Gaussian fit to the psychophysical data, and reliability as the inverse of its variance. Bias and reliability therefore correspond to the accuracy and variance of body-centered cues; that is, audio, visual, or audio–visual image motion that has been transformed into body-centered coordinates using an estimate of head rotation (see Figure 1). The weightings were based on the reliabilities (*rel_a_*, *rel_v_*) of the predicting conditions listed in Table 2:

(2) $$\begin{array}{c} rel_{a}=\frac{1}{\sigma_{a}^{2}}\;rel_{v}=\frac{1}{\sigma_{v}^{2}}\; \end{array}$$

(3) $$\begin{array}{c} w_{a}=\frac{rel_{a}}{rel_{a}+rel_{v}}\;w_{v}=\frac{rel_{v}}{rel_{a}+rel_{v}}\; \end{array}$$

It is important to note that the standard cue integration approach being referred to here is typically applied to situations where bias has been introduced externally via small cue conflicts (Alais & Burr, 2004b; Ernst & Banks, 2002; Landy et al., 1995). The explicit assumption in those papers is that the underlying signals themselves (i.e., the “estimators”) are unbiased, or “internally consistent” (Burge, Girshick, & Banks, 2010). This is not the case here, as the compensation process produces body-centered auditory and visual cues that are not necessarily unbiased; that is, the PSE describing these “predicting” conditions is not necessarily at a motion gain of 0 (perfect compensation). Nevertheless, the predicted precision for the cue-combined condition remains unchanged (see Scarfe & Hibbard, 2011). We return to the assumption of unbiased estimators when developing predictions for uncompensated image-motion cues (Model 3).

The standard cue integration approach also allowed us to predict the precision of the audio–visual cue (σ*_av_*). This is based on the variance of the respective auditory and visual predicting conditions (i.e., the reciprocal of the reliabilities defined in Equation 2):

(4) $$\begin{array}{c} \sigma_{av}=\sqrt{\frac{\sigma_{a}^{2}\sigma_{v}^{2}}{\sigma_{a}^{2}+\sigma_{v}^{2}}}\; \end{array}$$

#### Model 2: Body-centered integration, adjusted for shared noise

In the BCI model, the audio and visual cues are transformed into body-centered coordinates using the same self-movement signal. The transformed cues are therefore correlated because they share a common source of noise (σ*_sm_*). To account for this, the BCI model can be modified (BCI+) to include the correlation (ρ) between these cues (Oruç et al., 2003). This changes the way the reliabilities of auditory and visual cues are calculated:

(5) $$\begin{array}{c} \begin{array}{c} rel_{a}=1/\sigma_{a}^{2}-\left(\rho \sqrt{1/\sigma_{a}^{2}\times 1/\sigma_{v}^{2}}\right)\\ rel_{v}=1/\sigma_{v}^{2}-\left(\rho \sqrt{1/\sigma_{a}^{2}\times 1/\sigma_{v}^{2}}\right) \end{array}\; \end{array}$$

The augmented reliabilities can then be used to predict the audio–visual bias using Equations 1 and 3. The shared noise also changes the prediction for audio–visual precision, which can be calculated as follows:

(6) $$\begin{array}{c} rel_{av}=\frac{1/\sigma_{a}^{2}+1/\sigma_{v}^{2}-2\rho \sqrt{1/\sigma_{a}^{2}\times 1/\sigma_{v}^{2}}}{1-\rho^{2}}\; \end{array}$$

(7) $$\begin{array}{c} \sigma_{av}=\;\sqrt{\frac{1}{rel_{av}}}\; \end{array}$$

One approach to evaluating BCI+ is to fit the equations to the data by allowing the correlation (ρ) to be a free parameter. Note, however, that the correlation is defined as the ratio of the shared noise to the product of the noise associated with individual cues:

(8) $$\begin{array}{c} \rho =\;\frac{\sigma_{SM}^{2}}{\sqrt{\sigma_{a}^{2}\sigma_{v}^{2}}}\; \end{array}$$

We already know the variance of the individual cues ($\sigma_{a}^{2}$ and $\sigma_{v}^{2}$) from the psychometric functions of the predicting conditions. To estimate the variance of the self-movement signal ($\sigma_{SM}^{2}$) for each of the participants, we used the technique described by Haynes et al. (2024), described in Supplementary Appendix A. With $\sigma_{SM}^{2}$ now known, the goodness-of-fit of BCI+ can be directly compared to BCI, without having to correct for differences in the number of free parameters used.

#### Model 3: Image-centered integration

The above two models are based on auditory and visual motion that has been transformed into body-centered coordinates. As a comparison, we compared their predictions to a model based on uncompensated cues (i.e., auditory and visual image motion alone). The image-centered integration (ICI) model assumes that self-movement signals are ignored. To do this, note that performance in the auditory and visual conditions is limited by two sources of noise. Assuming these are Gaussian, we can use the variance sum law to isolate the precision of the image signals by subtracting the variance of the self-movement signal ($\sigma_{SM}^{2}$) from the respective auditory and visual predicting conditions:

(9) $$\begin{array}{c} \sigma_{a\_Im}=\;\sqrt{\sigma_{a}^{2}-\sigma_{SM}^{2}}\;\;\sigma_{v\_Im}=\;\sqrt{\sigma_{v}^{2}-\sigma_{SM}^{2}}\; \end{array}$$

We then used these to predict audio–visual precision using the equations defined in the BCI model.

As discussed above, models of cue combination typically assume that input signals such as auditory and visual image motion are unbiased, or internally consistent (Burge et al., 2010). On that basis, the ICI model predicts no auditory–visual bias or, put another way, an audio–visual PSE of 0 (i.e., veridical). To reiterate, the predicted precision for the ICI model is unaffected by any bias related to the signal inputs (see Scarfe & Hibbard, 2011).

### Across-trial noise analysis

At first sight, manipulating stimulus motion based on a proportion of ongoing self-movement, or motion gain, seems to resolve the problem that head movements vary both within and across trials. However, as discussed in Haynes et al. (2024), the judgment made by the participant is based on inputs coded as motion amplitude rather than motion gain. Variability in head movements at each level of stimulus gain also leads to unavoidable across-trial noise that is not accounted for by fitting a standard psychometric function. This additional across-trial noise can thus introduce surprising effects on the psychometric function, including a steeper slope than is fit by a standard cumulative Gaussian, and—at high head-movement variabilities—function asymptotes that deviate significantly from 0% and 100% that cannot be accounted for by the constrained lapse rates used in our standard analysis (Haynes et al., 2024). To address this issue, we therefore also fit the data with a custom psychometric analysis that captures this additional source of noise, and then re-fit the BCI, BCI+, and ICI models based on the result (see Supplementary Appendix B for full details). The goodness-of-fit of each model to the audio–visual condition data was calculated using RMSE.

### Statistics

Non-parametric bootstraps of 2000 samples were used to estimate standard errors around each psychometric function parameter using the PAL_PFML_BootstrapNonParametricMultiple function from the Palamedes toolbox in MATLAB, in order to construct CIs that were used to test for significant differences between empirical psychometric functions and predicted psychometric functions across each model in individual subjects. To construct CIs for the BCI+ and ICI models, bootstrapped psychometric function parameters were obtained for each of the three repetitions of the self-movement paradigm (Supplementary Appendix A). As for the empirical estimate, the variance sum law was used to extract the variance of self-motion signal ($\sigma_{SM}^{2}$) for each repetition, and the mean was taken as the final measure. Due to occasional extremes in sampling during the bootstrapping procedure, it was sometimes possible to obtain negative self-movement variances. This occurred when the bootstrapped sample produced self-movement precision that was greater than the precision of the audio and visual cues alone. These samples were excluded from calculation of the final bootstrapped self-movement variance parameter. Thus, it was possible for the final $\sigma_{SM}^{2}$ estimate to be based on the mean of one, two, or three repetitions. Of the 2000 bootstrapped estimates, this occurred 17.79%, 39.3%, and 39.2% of the time, respectively. Only 3.71% of the 2000 bootstrapped self-movement estimates were excluded entirely (i.e., when all three repetitions resulted in negative self-movement variances).

To analyze which model best predicted the data on a group level, repeated-measures ANOVAs with Model and Condition as factors were conducted on the squared errors from the model predictions and precision and bias, as well as on the RMSE values from the across-trial noise analysis. Significant effects were followed up with post hoc Bonferroni-corrected *t*-tests.

### Eye and head movement analysis

Eye movements were analyzed to ensure that participants maintained fixation on the fixation point. Eye movement analysis followed that of Haynes et al. (2024). Samples with less than 0.6 confidence (defined by Pupil Labs software) were excluded, and gaps were filled using linear interpolation (Halow, Liu, Folmer, & MacNeilage, 2023). The entire waveform was excluded from analysis if 50% or fewer samples remained. A Gaussian filter (σ = 16 Hz frequency domain) was used to smooth remaining waveforms, and velocity, acceleration, and jerk were established by taking the first, second, and third derivatives, respectively. The initial and final 20 samples of each waveform were removed. Saccades (and four samples either side) were removed from the analysis, with saccades detected using Wyatt's jerk analysis (jerk threshold = 20,000°/s^2^). Mean velocity and speed in the third head sweep were then calculated. For comparison, we calculated the eye velocity expected for compensatory vestibulo-ocular reflex (VOR) using the equation *E* = –*H*(1 + *R*/*D*) (Leigh & Zee, 2015), where *H* is the average head velocity per condition across participants, *R* = 0.1 m (the approximate distance from the eye to the center of head rotation), and *D* = 1.2 m (the distance from participant to speakers/LED ring). Eye movements could not be recorded from one participant due to technical problems.

Recorded head movements were smoothed using a MATLAB lowpass filter with a passband of 8 Hz. The temporal derivative was taken, and the median velocity was calculated over 20% to 60% of the third sweep length because we have previously shown that this region of interest produces a stable estimate (Haynes et al., 2024). The distribution of median velocities for each participant in each condition was then fit with a Gaussian to extract the mean and variance of head velocity.

### Data availability

Data and analysis codes are available on the Open Science Framework (https://osf.io/yj27m/).

## Results

### Psychophysics

#### Precision

**Figure 4.**
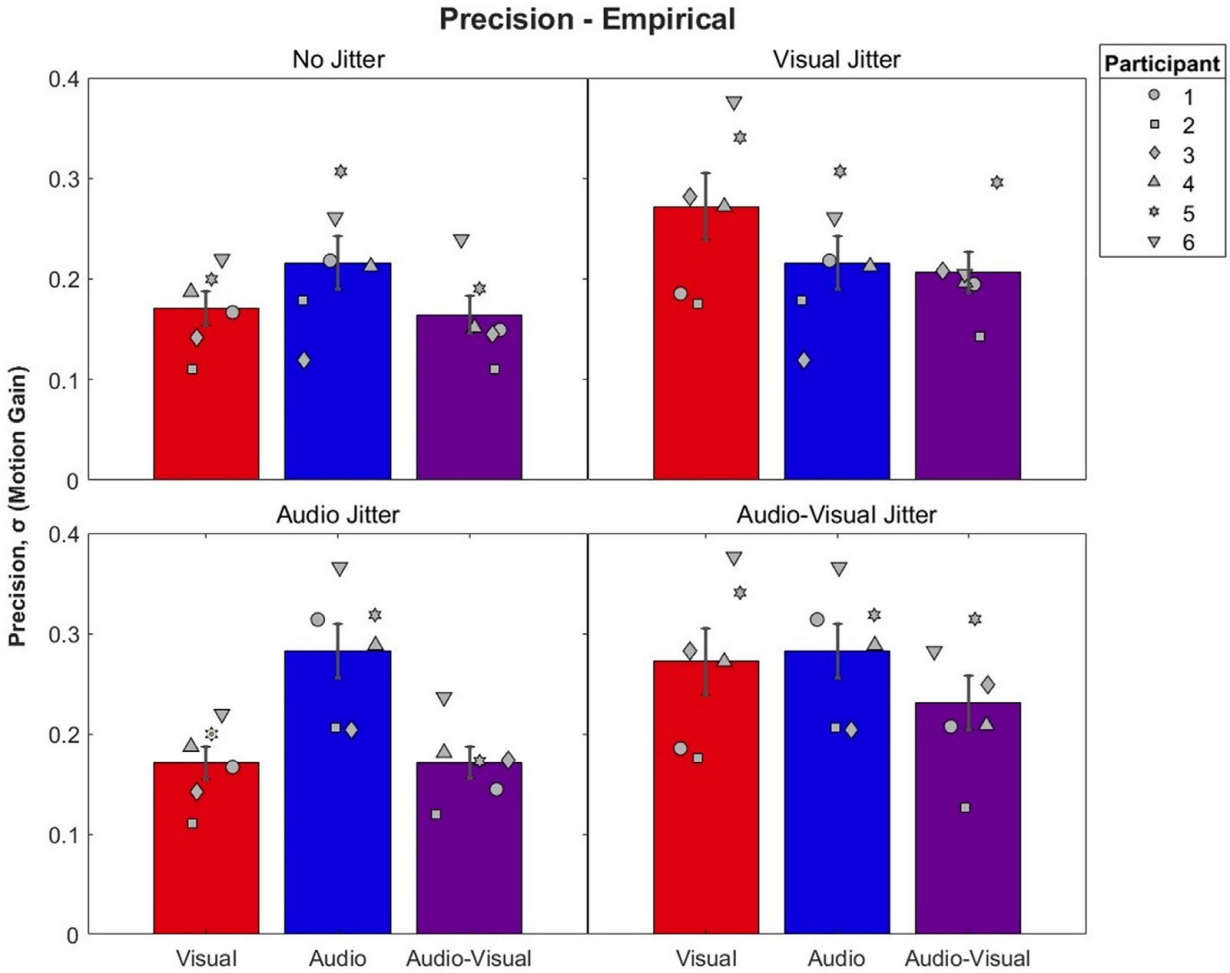
Mean and individual precision by condition. Audio–visual bars are presented alongside their predicting conditions, with visual and audio conditions indicated by red and blue, respectively. Note that predicting conditions can appear more than once across panels (Table 2). Error bars represent ±1 standard error between participants. Lower numerical values indicate smaller standard deviations and thus greater precision. Participants 5 and 6 were naïve to the hypotheses of the study.

Mean and individual precision for each condition can be seen in Figure 4. As expected, the addition of positional jitter decreased the precision of the cues to which it was applied, with the biggest decrease in precision occurring when applied to both cues, as indicated by larger standard deviations. Crucially, audio–visual precision was similar or better compared to the most precise audio or visual cue in each condition, with the most benefit of integration observed when both visual and audio signals had similar levels reliability in the audio–visual jitter condition.

**Figure 5.**
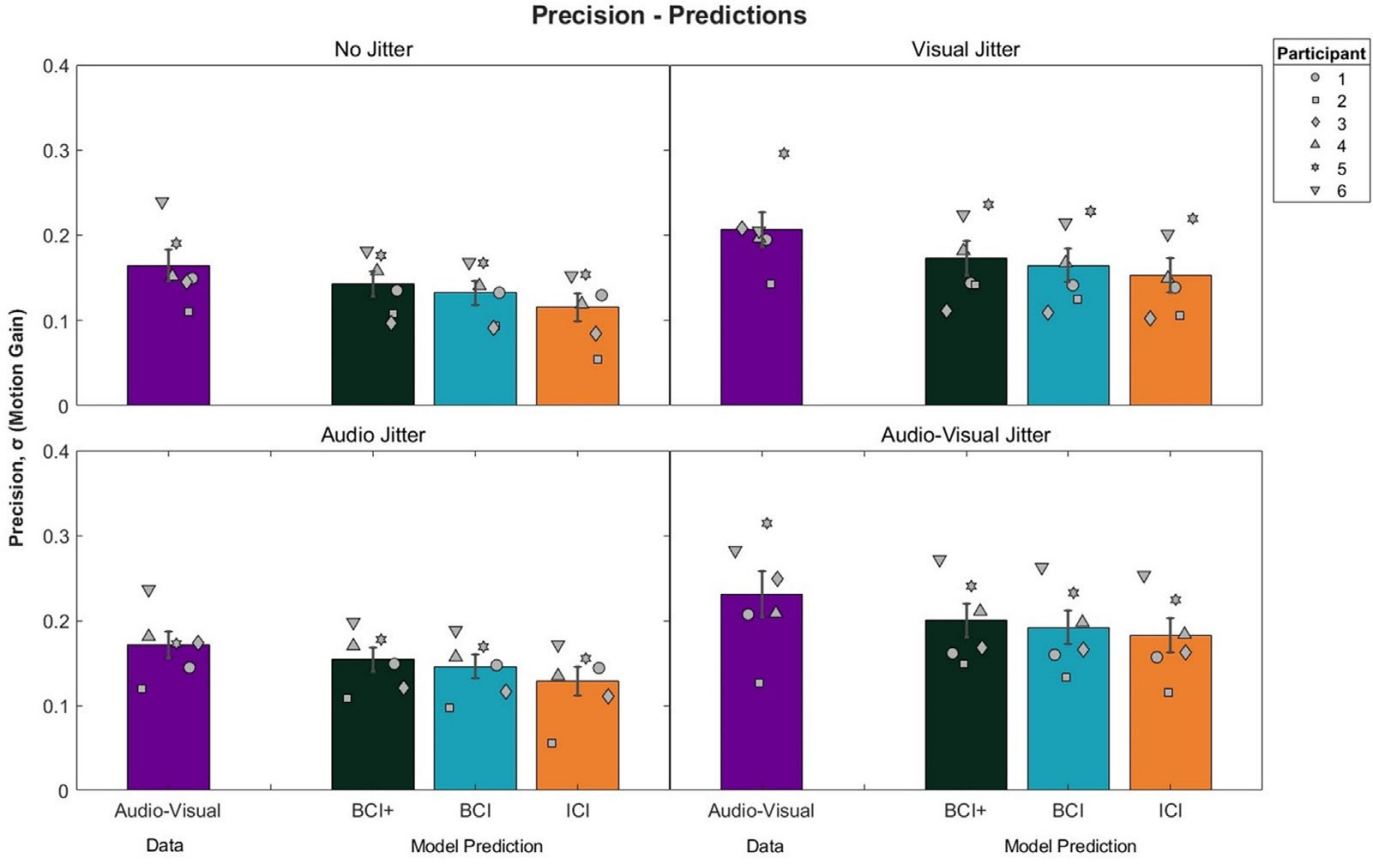
Audio–visual precision and model predictions by condition. Error bars represent ±1 standard error between participants. Lower numerical values indicate smaller standard deviations and thus greater precision. Participants 5 and 6 were naïve to the hypotheses of the study.

**Figure 6.**
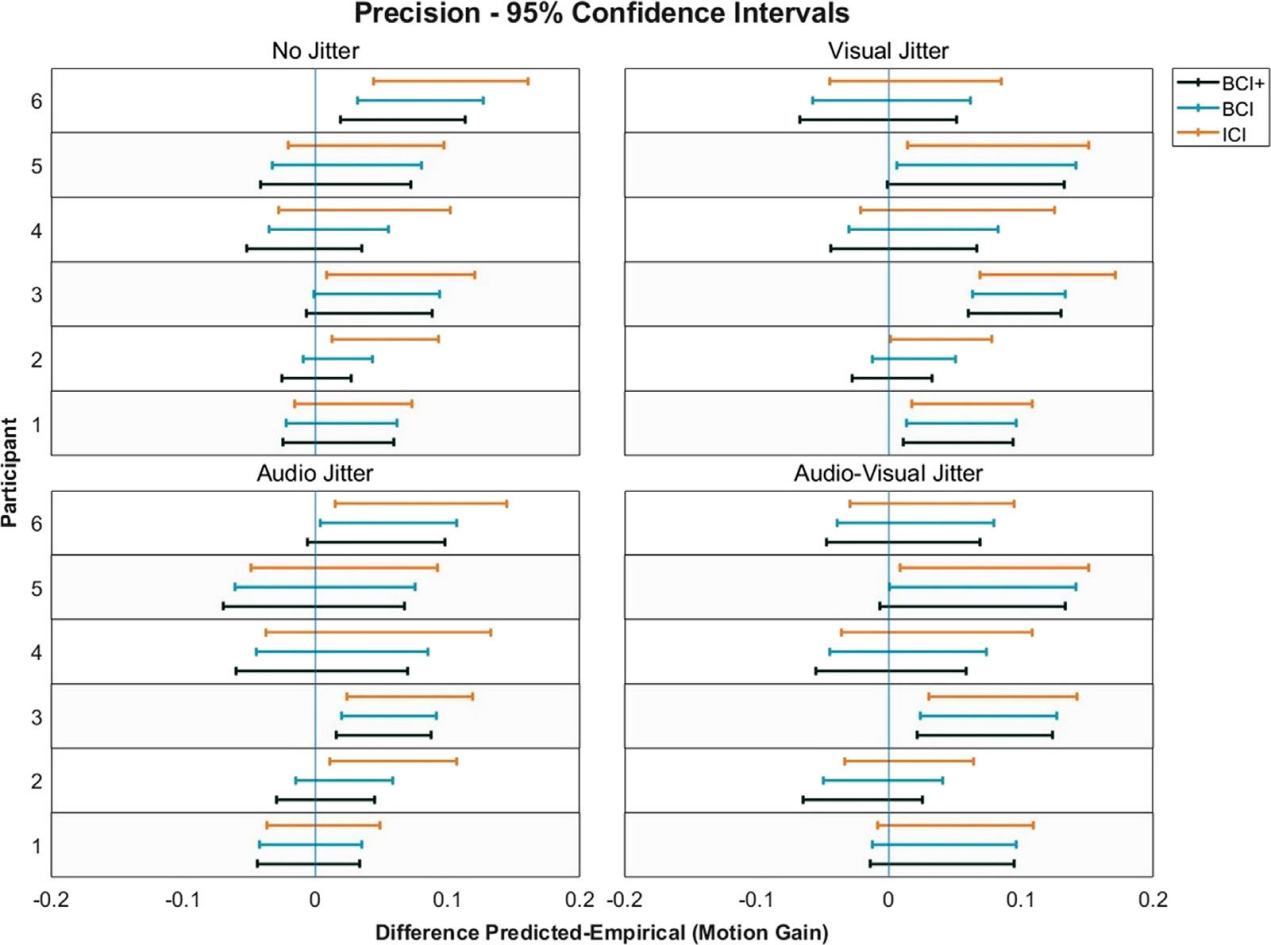
The 95% CIs for the difference between predicted and empirical audio–visual precision. The CIs that cross the 0 point indicate no significant difference between predicted and empirical precision. Participants 5 and 6 were naïve to the hypotheses of the study.

Model predictions compared to the audio–visual conditions can be seen in Figure 5. In general, the BCI+ model resulted in the closest prediction to the actual performance obtained for the audio–visual conditions. ICI predictions were the furthest from actual performance, suggesting that cue integration was based on some form of coordinate transform. A 3 × 4 repeated measures ANOVA was conducted on the squared differences between model predictions and observed precision (Table 3). A main effect of Model was found, *F*(2, 10) = 26.16, *p* < 0.001, η^2^*_G_*= 0.05. Bonferroni-corrected pairwise *t*-tests revealed that the BCI+ model was significantly better at predicting precision compared to the BCI model (average squared errors: BCI+ = 0.0017, BCI = 0.002, *p* = 0.001) and to the ICI model (average squared errors: ICI = 0.003, *p* < 0.001). The BCI model was also better at predicting precision compared to the ICI model (*p* < 0.001). No other main effects or interactions were significant; for Condition, *F*(3, 15) = 0.83, *p* = 0.50, and for Model × Condition, *F*(6, 30) = 0.73, *p* = 0.63. It is also worth noting that all three models predicted greater precision than actually observed, an important point taken up in the Discussion. On an individual level, CIs revealed that the BCI model predicted precision in 16 of 24 cases, whereas the ICI model predicted precision in 12 of 24 cases. The BCI+ model again had the best performance, predicting individual precision in 19 of 24 cases (Figure 6).

**Table 3.** Mean ± *SD* squared errors for precision by condition and model.

Condition	ICI	BCI	BCI+
No jitter	0.0029 ± 0.0026	0.0015 ± 0.0020	0.0010 ± 0.0015
Visual jitter	0.0040 ± 0.0040	0.0031 ± 0.0037	0.0027 ± 0.0035
Audio jitter	0.0024 ± 0.0020	0.0011 ± 0.0013	0.0008 ± 0.0011
Audio–visual jitter	0.0033 ± 0.0036	0.0027 ± 0.0032	0.0024 ± 0.0029

#### Bias

**Figure 7.**
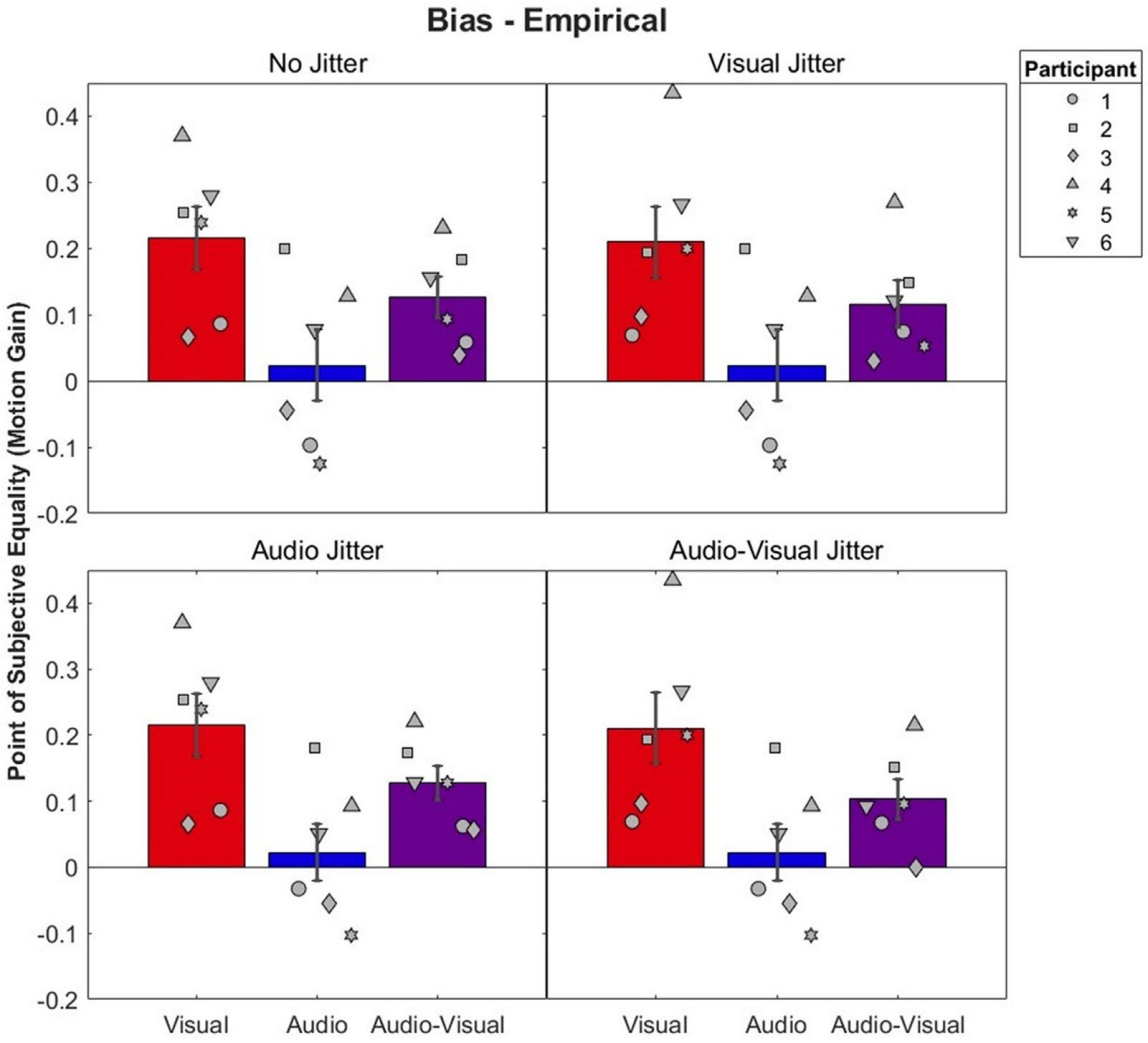
Mean and individual biases by audio–visual condition. Audio–visual bars are presented alongside their predicting conditions, and visual and audio conditions are indicated by red and blue, respectively. Note that predicting conditions can appear more than once across panels (Table 2). Error bars represent ±1 standard error between participants. Participants 5 and 6 were naïve to the hypotheses of the study.

Mean biases for each condition can be seen in Figure 7. Biases were largest in all visual conditions, indicating that participants perceived a stationary visual stimulus to move in the opposite direction of the head movements (i.e., a Filehne illusion) (Filehne, 1922; Freeman, 2007; Haarmeier & Thier, 1996; Mack & Herman, 1973). As expected, audio–visual biases were in between visual and audio biases.

**Figure 8.**
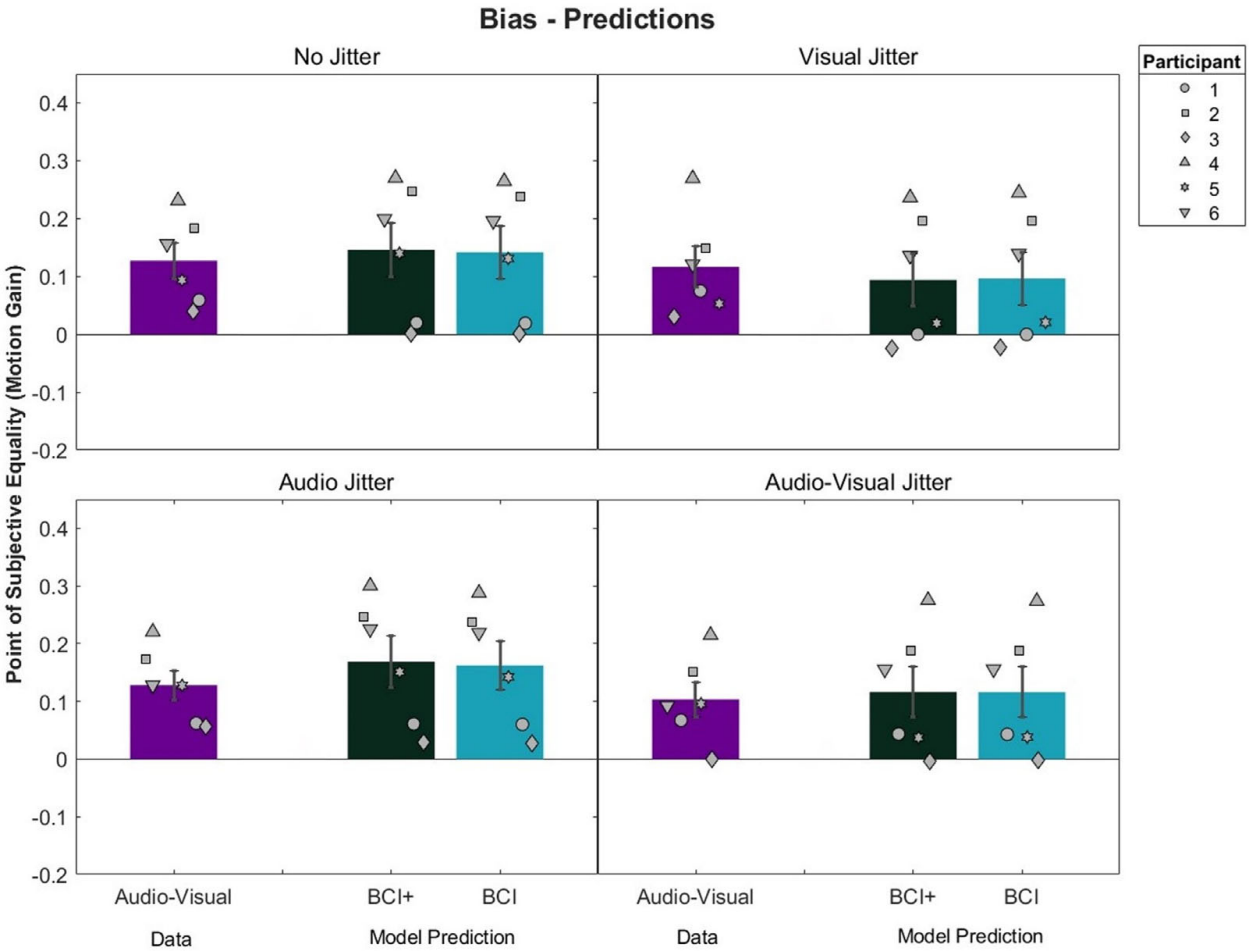
Audio–visual bias and model predictions by condition. Error bars represent ±1 standard error between participants. Participants 5 and 6 were naïve to the hypotheses of the study.

**Figure 9.**
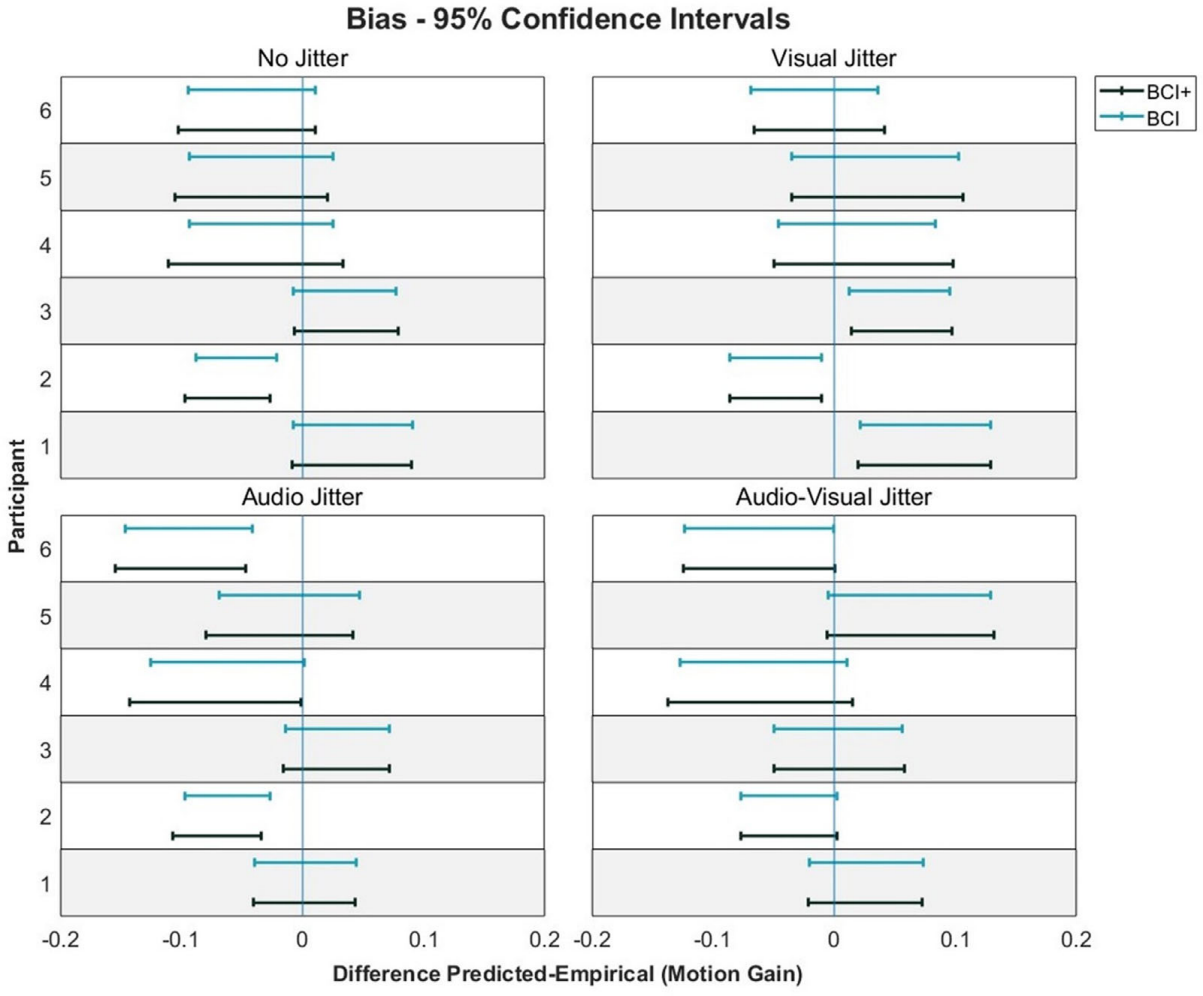
The 95% CIs for the difference between predicted and empirical audio–visual precision. Participants 5 and 6 were naïve to the hypotheses of the study.

Model predictions compared to the audio–visual conditions can be seen in Figure 8. Recall that the ICI model, by definition, predicts a bias of 0 and so is not included in the figure. In general, both models predicted similar biases. A 2 × 4 repeated-measures ANOVA on the squared differences between model predictions and observed biases was conducted (Table 4). This analysis revealed a significant main effect of Model, *F*(1, 5) = 7.63, *p* = 0.04, η^2^*_G_* = 0.006, with lower squared errors for the BCI model (mean = 0.022) versus the BCI+ model (mean = 0.0026). No main effect of Condition was found, *F*(3, 15) = 0.47, *p* = 0.71. A significant interaction between Model and Condition was found, *F*(3, 15) = 3.97, *p* = 0.03, η^2^*_G_* = 0.005, with lower squared errors for the no-jitter and audio-jitter conditions for the BCI model, and similar squared errors across both models in the visual and audio–visual jitter conditions. However, Bonferroni-corrected pairwise *t*-tests revealed no significant differences across any model or condition. On an individual level, both models predicted biases in 17 of 24 cases (Figure 9). There was no consistent pattern in whether models over- or underestimated biases.

**Table 4.** Mean ± *SD* squared errors for biases by condition and model.

Condition	BCI	BCI+
No jitter	0.0017 ± 0.0007	0.0021 ± 0.0010
Visual jitter	0.0021 ± 0.0020	0.0022 ± 0.0019
Audio jitter	0.0030 ± 0.0032	0.0037 ± 0.0039
Audio–visual jitter	0.0021 ± 0.0017	0.0022 ± 0.0017

### Eye and head movements

**Figure 10.**
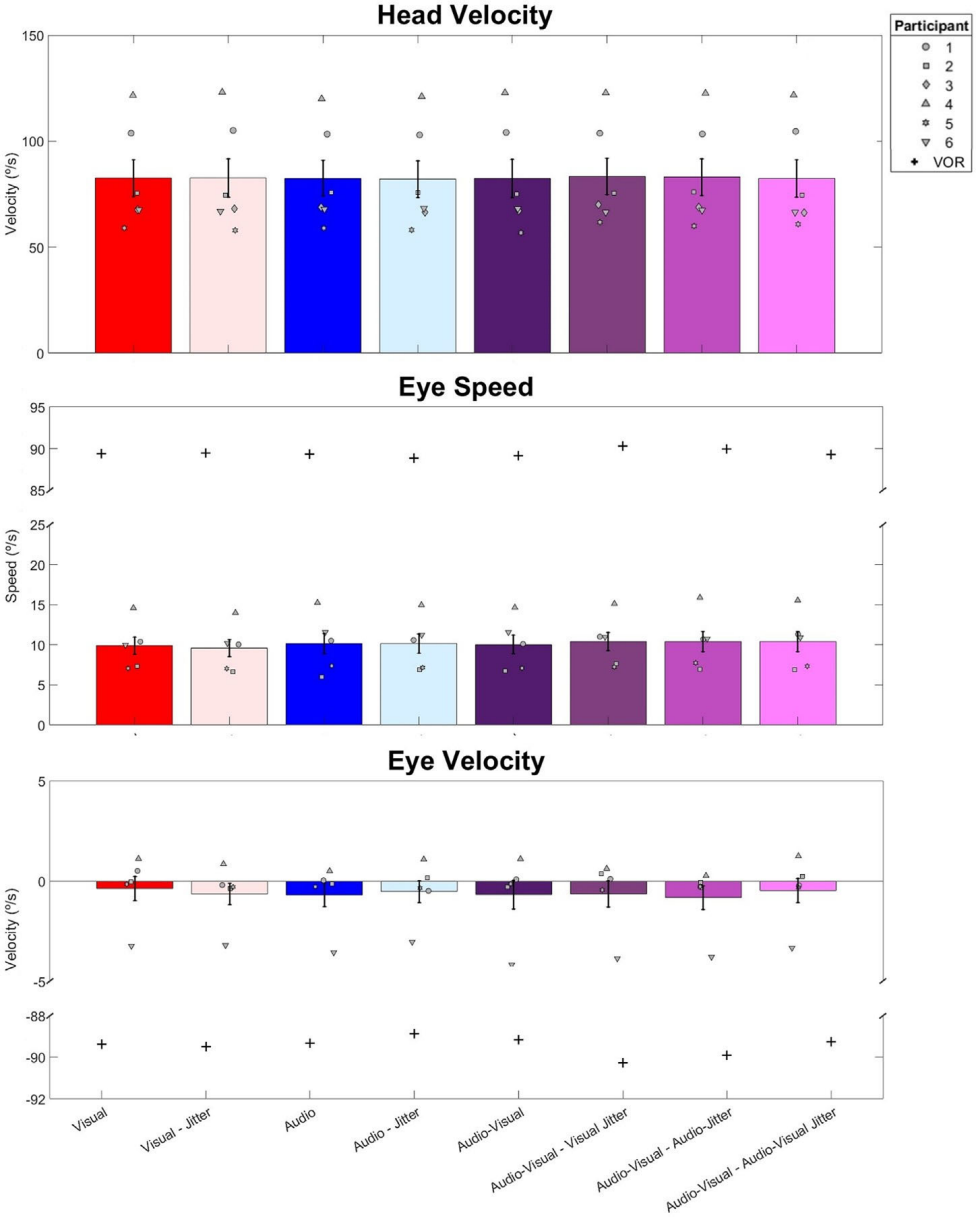
(Top) Absolute head movement velocities across all six participants in each condition. (Middle) Eye movement speeds for five of the six participants. (Bottom) Eye movement velocities for five of the six participants. Negative velocities indicate that the eyes moved in the opposite direction to the head. Bars show means across participants, and error bars represent ±1 standard error between participants. The plus symbols (+) indicate compensatory VOR. Participants 5 and 6 were naïve to the hypotheses of the study.

As can be seen in Figure 10, head movement velocities within participants were similar across all eight conditions of the main task, although speeds across participants varied. Importantly, eye movements were much smaller than would have been expected from the VOR (+ symbols in Figure 10) needed to perfectly compensate the observed head movements, suggesting that participants were able to keep their eyes fixed in the head throughout the experiment.

### Across-trial noise analysis

**Figure 11.**
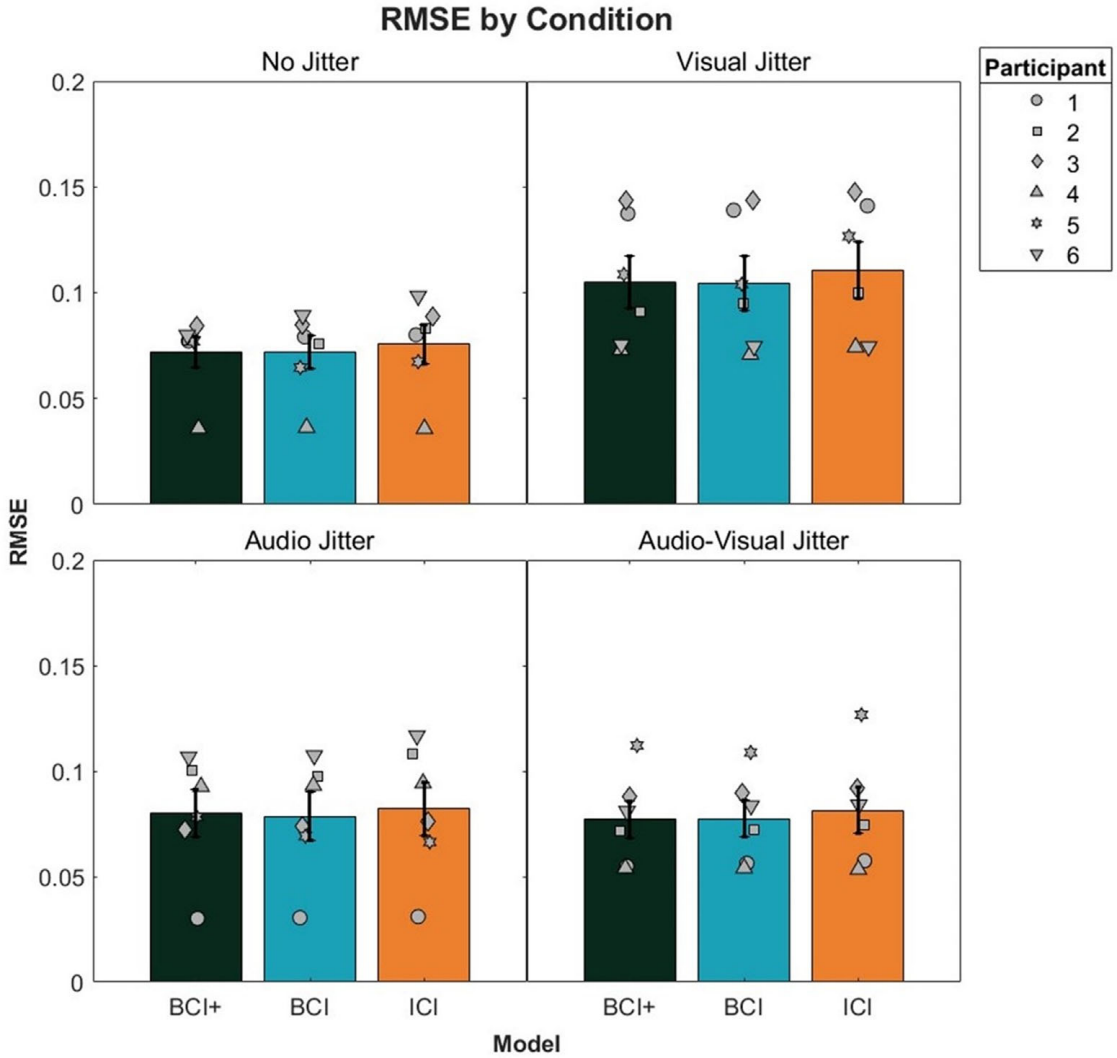
RMSE for model fits to each audio–visual condition. Error bars represent ±1 standard error between participants. Participants 5 and 6 were naïve to the hypotheses of the study.

Mean and standard error RMSEs for each model and audio–visual condition can be seen in Figure 11. In general, the BCI and BCI+ models were similar across conditions, but both of these models were better than the ICI model in all conditions. A 3 × 4 repeated-measures ANOVA with Model and Condition as factors was conducted on the RMSEs. The main effect of Model was significant, *F*(2, 10) = 6.49, p = 0.02, η^2^*_G_* = 0.007. Bonferroni-corrected paired *t*-tests revealed significant differences between the BCI+ and ICI models (average RMSE: BCI+ = 0.084, ICI = 0.087, *p* = 0.03) and the BCI and ICI models (average RMSE: BCI = 0.083, *p* = 0.005). No significant difference was found between the BCI+ and BCI models. No other main effects or interactions were significant; for Condition, *F*(6, 30) = 1.95, *p* = 0.16, and for Model × Condition, *F*(6, 24) = 0.24, *p* = 0.68, with Greenhouse–Geisser correction for sphericity. On an individual level, the BCI+ model was the best-fitting model in 14 of 24 cases, although in general the difference in RMSE between the BCI and BCI+ models was small.

Although this “across-trial noise” analysis appears to show less difference between BCI and BCI+ models, compared to the more standard analysis examined earlier, it is important to note that these two approaches differ markedly in how the psychometric function parameter predictions are assessed. In particular, the more standard analysis considers predictions based on precision and bias separately, whereas the “across-trial noise” analysis shown in Figure 11 groups these two parameters together. Moreover, conclusions are drawn about the latter on the basis of the goodness of fit of model-driven psychometric functions to the raw psychophysical data, as opposed to comparing empirical and model bias and precision in the case of the more standard model. Given that the more standard analysis showed minimal differences in bias predictions across models (e.g., Figure 8), we suspect that this is the reason why we found smaller differences between them when using the “across-trial noise” analysis.

Overall, both approaches suggest that integration is based on compensated cues, given the better performance of the BCI and BCI+ models in comparison to the ICI model. Moreover, the more standard approach shows that audio–visual precision is best accounted for by the BCI+ model, at both a cohort and an individual level.

## Discussion

The integration of sensory signals is made complicated during self-movement because cues may be represented in different coordinate frames, and are subsequently affected differently by head and/or eye movements. We proposed that integration of audio and visual signals occurs following the transformation of these cues into a common coordinate frame. Participants completed a psychophysical multisensory integration task while making active yaw head rotations and fixating on a head-fixed target. Participants judged whether a visual, auditory, or audio–visual target, presented at high or low stimulus reliabilities, moved left or right. We found that performance in the audio–visual conditions was well-described by models based on the combination of compensated audio and visual signals represented in a common coordinate frame. A model based on uncompensated image-based signals could not explain performance in the majority of audio–visual conditions in most participants. We also found that taking into account the inevitable shared noise between compensated cues accounted for the increase in audio–visual precision better than other models. Accordingly, our data show that it is likely that audio and visual cues are first transformed into common coordinates before these signals are integrated according to principles of optimal integration.

Previous studies have considered the integration of moving audio–visual targets in stationary observers and consistently found evidence for optimal integration (Alais & Burr, 2004a; Meyer & Wuerger, 2001; Soto-Faraco et al., 2004; Wuerger et al., 2010). For example, variability in the estimate of moving object arrival times is reduced when both audio and visual cues are present, consistent with maximum likelihood principles (Wuerger et al., 2010). Similarly, detection thresholds for motion perception are significantly improved in the presence of audio–visual stimuli together, rather than individual modalities alone (Alais & Burr, 2004a). By contrast, few studies have considered the impact of self-motion on the process of integration, although several studies have demonstrated that self-movement can impact the perception of auditory and visual stimulus motion more generally. For example, rotation and translation of the body and/or head can impact the localization of both auditory and visual targets (Carriot, Bryan, DiZio, & Lackner, 2011; Cooper, Carlile, & Alais, 2008; Lackner & DiZio, 2010; Teramoto, Cui, Sakamoto, & Gyoba, 2014), and stationary stimuli are perceived as moving in the opposite direction to head and/or eye movements (Freeman, 2007; Freeman et al., 2017). This latter so-called Filehne illusion is also present in our current study, reflected in the biases obtained for audio, visual, and audio–visual conditions. Curiously, although we found visual biases similar to those previously reported for smooth eye pursuit (Filehne, 1922; Furman & Gur, 2012), the auditory biases were much smaller than in an earlier study from our lab that used similar auditory stimuli (Freeman et al., 2017). The smaller biases may have arisen because here we presented the cues to be judged during a single sweep of the head movement, instead of continuously as in Freeman et al. (2017). We note, too, that the Filehne illusion depends on basic stimulus properties such as spatial frequency (Freeman & Banks, 1998; Wertheim, 1987), which determine the size of the image-motion estimate to which the self-motion signal is compared. Hence, the Filehne illusions found for the visual and auditory conditions will also depend on the specific auditory and visual stimuli used. As such, it is possible that we found a larger visual versus auditory Filehne illusion in the present study due to differences in stimulus parameters, such as the standard deviation of the stimuli. Our results build upon existing literature, proposing a mechanism through which self-movement, visual motion, and auditory motion are integrated to help us perceive movement.

In our study, participants maintained their gaze on a head-fixed target while making yaw head movements, meaning that both the eyes and ears moved with respect to the external world. Accordingly, to successfully make directional judgments as in the present task, observers had to account for this self-movement and put audio and visual cues into a common reference frame prior to integration. We suggest that the common coordinate frame is body centered, given that this reference frame remains stationary during head and eye movements, and is parsimonious, given the evidence both here and in other papers that both auditory and visual signals can be transformed into this reference frame (Furman & Gur, 2012; Goossens & van Opstal, 1999; Kopinska & Harris, 2003; Lewald et al., 1999). Yet it is possible that there is an alternative route to cue integration. In particular, previous research on auditory localization has suggested that auditory signals are first transformed into an eye-centered reference frame, and then integrated with visual signals before a transformation to body-centered coordinates at a later stage (Lee & Groh, 2012; Lewald, Dörrscheidt, & Ehrenstein, 2000). The present data cannot differentiate among these alternate transformation routes, given that eye- and head-centered reference frames coincide due to the type of head movement employed in the study. However, it is important to note that, first, the eye-centered model has been used to explain the influence of eye gaze on auditory localization alone, rather than audio–visual cue combination as explored here, and, second, the eye-centered model is based on localization of auditory cues in stationary observers rather than in cases when the head and eyes move. In addition, as we pointed out in the Introduction, there is good evidence that localization and gaze orienting to auditory and visual targets are driven by body-centered not eye-centered coordinates (Goossens & van Opstal, 1999; Kopinska & Harris, 2003). Moreover, evidence suggests that numerous coordinate frames are represented at different neural levels. For example, auditory and visual motion may be represented in eye-centered, head-centered, or even hybrid frames across regions, including the superior and inferior colliculus, primate ventral intraparietal cortex, and V1 (Bulkin & Groh, 2006; Furman & Gur, 2012; Ilg, Schumann, & Thier, 2004; Ilg & Thier, 1996; Lee & Groh, 2012; Zhang, Heuer, & Britten, 2004). Future research is therefore necessary to delineate precisely which coordinate frame transforms are conducted during multisensory integration and in what order. Although we propose a body-centered model, many alternatives are possible, given the diversity of neural representations and sensory tasks implicating optimal integration.

Outstanding questions remain regarding which sensory signals are used to compensate for self-movement in our paradigm. It is likely that self-movement compensation would include cues from the vestibular system, neck proprioception, efference copy, and signals from eye muscles (Dokka, MacNeilage, DeAngelis, & Angelaki, 2015; Furman & Gur, 2012; Genzel et al., 2016). The role of the eye muscles and efference copy has been widely explored in relation to visual localization during smooth pursuit eye movements (Bogadhi, Montagnini, & Masson, 2013; Furman & Gur, 2012). Similarly, visuo-vestibular integration is necessary to compensate for translational self-movement (Dokka et al., 2015), whereas proprioceptive and vestibular signals are required for auditory spatial updating (Genzel et al., 2016). In our research, we considered a unified “self-movement” signal as the source for self-movement compensation, without distinguishing which inputs formed this signal. As such, future research should aim to more precisely define which sensory modalities form this self-movement signal and under which circumstances they are combined.

Our results and analysis suggest that precision in the audio–visual conditions was not completely optimal. As expected, we observed the greatest increase in audio–visual precision when both audio and visual cues had similar reliabilities. However, when audio and visual reliabilities diverged, audio–visual precision was close to the “best” unimodal condition. In general, non-jittered visual signals were more precise than auditory signals. Accordingly, it may be difficult to establish whether participants used a “best cue” strategy rather than an optimal integration strategy. However, the clearly increased precision in the audio–visual jittered condition would suggest that participants did indeed engage in (near) optimal integration. Given that all trial types were interleaved, it seems unlikely that participants would switch between these alternative strategies on a trial-by-trial basis. Nonetheless, to further assess whether and how much audio–visual integration deviates from optimality and to better distinguish between a “best cue” versus an optimal integration strategy, future research may be necessary. Such research could individually tailor audio and visual precision to more clearly explore the increased precision apparent in audio–visual conditions or introduce cue conflicts to examine whether predicted and measured audio and visual weightings diverge (Rohde, van Dam, & Ernst, 2016).

Finally, the models presented here predicted greater precision than was actually observed, suggesting an additional unaccounted source of noise. Although we emphasized the conversion of signals into a common *reference frame*, it is likely that further conversions are necessary to transform audio and visual signals into common *units*. When observing moving objects, visual cues are dominated by speed (Freeman, Cucu, & Smith, 2018; Reisbeck & Gegenfurtner, 1999), while auditory cues are dominated by displacement (Carlile & Best, 2002; Freeman, Leung, Wufong, Orchard-Mills, Carlile, & Alais, 2014). Thus, when combining hearing and vision, auditory and visual cues must also be transformed into common units (i.e., displacement to speed, or speed to displacement). This process likely adds noise (Haynes et al., 2024). As we predicted audio–visual conditions on the basis of separate audio and visual conditions, it is possible that this unit conversion noise is missing from our final predictions. After all, the need for common units is only necessary when hearing and vision are directly compared to each other, which in our experiments corresponds to the audio–visual conditions, not the predicting conditions. Furthermore, when the eye and/or head is moving, an additional unit conversion is needed between self-movement and image-motion signals. Vestibular and motor signals are likely to be encoded in terms of acceleration and speed units (Angelaki & Cullen, 2008; Cullen, 2019; Freeman et al., 2018), making the combination of self-movement and visual motion relatively straightforward. However, additional conversion is needed to combined speed-based self-movement cues with displacement-based auditory cues. Given that our predicting conditions involved the same self-movement as the combined audio–visual conditions, we have likely accounted for the unit conversion noise for each modality separately. However, it is possible that the *shared* noise from these conversions remains unaccounted for. Accordingly, future experiments and models are necessary to resolve the unit conversion problem, over and above the reference frame issue we present here.

## Conclusions

Overall, here we have demonstrated that audio–visual motion perception during active self-movement is based on the combination of sensory signals, which are transformed into a common coordinate frame. Importantly, our study investigated audio–visual integration during self-generated, active movements, expanding our knowledge of multisensory integration to more naturalistic task constraints. We propose that the common coordinate frame is body centered; however, alternatives may be possible based on the modalities and tasks involved in any given multisensory scenario. Accordingly, this research opens a new avenue for future work to investigate integration during natural, active self-movement.

## Supplementary Material

Supplement 1
