# Supplementary material for: A model of audio–visual motion integration during active self-movement
Source: J Vis. 2025 Feb 19;25(2):8. doi: 10.1167/jov.25.2.8 (PMC11841688; doi:10.1167/jov.25.2.8)
Supplement: Supplement 1 [file jovi-25-2-8_s001.docx]

**Appendix A: Measuring Self-Movement Variability**

A separate paradigm was used to measure the precision of self-movement signals, based on Haynes et al. (2024). The precision of self-movement was required to calculate the shared noise between the audio and visual signals for the BCI+ model prediction (see Equation 8). All participants from the main paradigm completed this additional paradigm in a separate session after completing the main experiment. We measured speed discrimination in a separate 2-interval forced choice (2IFC) task with two phases: one containing a self-movement signal and one without this signal. Briefly, in Phase 1, the standard interval consists of the participant moving the head while head-fixed visual stimulus appears ‘on the nose’ (and consequently does not generate ‘image’ motion across the retina). The test interval consists of the same visual stimulus that is scaled by a motion gain, but presented while the head is stationary. The participant is then asked in which interval the stimulus appears to ‘move more’, and a psychometric function is obtained. In Phase 2, the visual stimuli from Phase 1 are replayed, but the head remains stationary throughout. Accordingly, we assume that the slope of psychometric function in Phase 1 is limited by two sources of noise, one corresponding to the self-movement signal and the other the image-motion signal, while Phase 2 is limited by noise from the image signal alone. Using the variance sum law, the precision of self-movement signals can therefore be estimated by subtracting half of the variance of Phase 2 from Phase 1.

***Procedure***

Participants were first trained to move their head at the average speed they moved in the main paradigm. A red light moved sinusoidally with the velocity and amplitude of each participants’ head movements, measured in the main paradigm. Participants had to memorise the motion of this light, and then replicate it by moving their head with a red light yoked to their head movements. Trials were classed as correct if they fell within ±10% of the target speed. Participants repeated the training at least three times, until 75% of trials were correct. We have shown previously that this type of training paradigm can be used to enable individual participants to reproduce a wide range of different average head speeds (Haynes et al., 2024).

The task consisted of a 2IFC, repeated in two phases. In Phase 1, Interval 1, participants made back-and-forth yaw head movements at the trained speed. On the third head sweep, a green target light was yoked to the head movement. In Phase 1, Interval 2, participants kept their head stationary while the target light was replayed and scaled to a proportion of the original head speed. Participants then reported whether the target moved faster in the first or second interval. A cumulative Gaussian psychometric function was fitted to response data of Phase 1, with the Point of Subjective Equality used to determine the speed of the targets in Phase 2. In Phase 2, both intervals from Phase 1 were replayed with the participant remaining stationary throughout. In Interval 1, target speed was presented at the PSE from Phase 1. Interval 2 targets were scaled to a proportion of the Interval 1 target speed. Participants again had to judge which interval contained the faster target. The entire procedure was repeated three times in total.

***Stimuli and Equipment***

Stimuli were presented with the same LED ring as the main paradigm. Eye and head movement were collected using the same equipment as the main paradigm.

The target light was a diffuse green LED blob spanning approximately 2.25°. The target light was yoked to the participants’ head movements in Phase 1, Interval 1. In Phase 1, Interval 2, the target light moved on the same path determined by the Interval 1 head movements, but its velocity was scaled as a proportion of head speed, from 40-100% of head speed in seven steps.

In Phase 2 the targets moved on the same path as Phase 1, Interval 1, determined by the head movement. Target velocity in Interval 1 was scaled to match the perceived speed of the target, determined by the Point of Subjective Equality (PSE) from Phase 1. Interval 2 target velocity was the PSE ± 30% in seven steps.

In both phases, each target velocity was repeated 10 times, and the entire paradigm was repeated three times, giving a total of 210 trials per phase (7 target velocities * 10 presentations * 3 repetitions). The entire paradigm took approximately 1 hour to complete.

**Data Analysis**

***Psychometric Analysis***

Data were analysed in MATLAB r2022b. The proportion of trials in which the participant responded the target was faster were calculated for each target velocity. For each repetition of Phase 2, target velocities were normalised to 0±30% of head speed by subtracting the PSE. Cumulative Gaussian psychometric functions were fitted using the Palamedes Toolbox, using the PAL_PMFL_FIT function. PSE, slopes, and lapse rates were free parameters, with lapse rates constrained to values between 0-0.02 (Prins, 2012). The PSE was defined as the 50% point of the psychometric function. The precision was defined as the inverse of the psychometric function slope (i.e., the standard deviation of the cumulative Gaussian fit to the data), such that larger numerical values indicated poorer precision.

The variance of the self-movement signal was calculated from the precision values obtained from Phase 1 (*Ph1*) and 2 (*Ph2*):

$$\sigma_{SM}^{2}= \sigma_{Ph1}^{2}-\frac{\sigma_{Ph2}^{2}}{2}$$

Note that the precision values of Phase 2 are halved, as both intervals contain image noise, whereas Phase 1 only contained image noise during the test interval. The self-movement variance was calculated for each repetition, and the square-root of the average variance used to estimate self-movement precision for the BCI+ model.

Estimates of the variability around parameter estimates were calculated as described for the main experiment, using 2,000 nonparametric bootstrapped samples with the function PAL_PFML_BootstrapNonParametricMultiple.

Note that this analysis is consistent with the standard psychophysical approach used in the main text, whereas Haynes et al. (2024) used an analysis consistent with the Across-Trial Noise analysis outlined in Appendix B. As noted in the main text, there is little difference between these two approaches.

***Head and Eye Movement Analysis***

Head and eye movements were analysed in the same way as the main experiment. Eye movement data for one participant was not recorded due to technical problems.

**Results**

| Table 1  Self-Movement Variability Results | | | | |
| --- | --- | --- | --- | --- |
| Participant | Phase 1 PSE (Motion gain) | Self-Movement Precision  (Motion gain) | Head Movement Velocity (°/s) | Eye Movement Velocity (°/s) |
| 1 | 0.64 | 0.038 | 123.23 | -0.49 |
| 2 | 0.73 | 0.094 | 64.16 | -0.20 |
| 3 | 0.78 | 0.046 | 57.49 | N/A |
| 4 | 0.72 | 0.104 | 88.78 | -0.01 |
| 5 | 0.60 | 0.086 | 113.99 | -2.75 |
| 6 | 0.69 | 0.099 | 71.30 | 0.20 |

Results can be seen in Table 1. Phase 1 PSEs for all participants were < 1, indicating that stimuli pursued by the head were perceived as slower than those that were not pursed. I.e., a PSE of 0.7 indicates that a stimulus moving past a stationary participant has to be slowed by 30% to be perceived as moving at the same speed as during the pursuit interval. This effect thus resembles a classic Aubert-Fleischl effect (Aubert, 1886; Dichgans et al., 1975; Garzorz et al., 2018), driven by head movements rather than eye pursuit. Similar findings are reported by Haynes et al. (2024).

Two participants had negative self-movement variance estimations on at least one repetition of the paradigm. These repetitions were excluded from the final average estimate of self-movement variances. The square root of the precisions reported in Table 1 (i.e., the self-movement variance) were used to generate model predictions in the main experiment.

Head movement velocities were similar to those obtained in the main experiment, with the exception of participant 5 whose head speeds were consistently faster compared to the main experiment. Eye movement velocities were negligible, indicating that participants were successfully able to track the head-fixed fixation point.

**Appendix B: Across-Trial Noise Analysis**

**Unimodal conditions**

On each trial, auditory or visual stimuli move across the speaker/ LED array. Stimulus motion ($M$) is made a fixed proportion ($g$) of the recorded head rotation ($H$) in real time:

$M\left( t \right)=gH\left( t \right)$ (1)

We refer to $g$ as the ‘motion gain’. When $g$ = 1, the stimulus moves at the same speed and direction as the head rotation i.e. on the nose. When $g$ = 0 , the stimulus is stationary. When $g$ = -1, the stimulus moves at the same speed but in the opposite direction to the head.

In both the unimodal and bimodal conditions, the task for observer is to judge whether $M$ moved to the left or right of the body during the head rotation made in the 3^rd^ sweep. For hearing, $M$ is the sum of image motion ($I$) and head rotation ($H$). In our experiment, this is also the case for vision because we inhibit eye rotations in the skull by providing a head-centred fixation target (akin to a scratch on a pair of glasses, albeit at a more comfortable viewing distance!). Eye movement analysis shown in Figure 10 indicates that they are able to do this very well, which confirms the finding of Haynes et al. (2024). Hence for both modalities:

$M=I+H$ (2)

To recover the body-centred motion $M$, the observer must estimate $I$ and $H$ from internal signals $i$ and $h$. We assume both are corrupted by fixed Gaussian noise with 0 mean. Using $N\left( \mu,\sigma\right)$ to denote a normal distribution with mean $\mu$ and standard deviation $\sigma$, then across trials:

$i=\mu_{i}+N\left( 0,\sigma_{i} \right)$ (3)

$h=\mu_{h}+N\left( 0,\sigma_{h} \right)$ (4)

Head movements will vary across trials, which means $h$ must vary with this too. The mean $\mu_{h}$ is therefore a random variable. This is also the case for $i$ because stimulus motion is yoked to the head movement as defined by Eqn (1). Assuming $H$ is also Gaussian distributed across trials, and noting that $I=\left( g-1 \right)H$ from Eqns (1) and (2):

$i=b_{i}(g-1)N\left( \mu_{H},\sigma_{H} \right)+N\left( 0,\sigma_{i} \right)$ (5)

$h=N\left( \mu_{H},\sigma_{H} \right)+N\left( 0,\sigma_{h} \right)$ (6)

where $b_{i}$ is a bias term that sets the gain of the image-movement signal relative to its input i.e. $i=b_{i}I$. Note the bias term also captures the relative difference in accuracy between the head movement signal and image-movement signal. Although more general accounts include a bias term to $h$ (see, for instance, Freeman & Banks, 1998), if we assume linearity as we do here, perceived motion would be determined by the ratio of these two bias terms. This is equivalent to the single parameter $b_{i}$ (see Freeman, 2001, for a comparison between linear and non-linear accounts).

Combining (5) and (6) with (2), perceived motion ($m_{i}$) is given by:

${m_{i}=(b}_{i}\left( g-1 \right)+1)N\left( \mu_{H},\sigma_{H} \right)+N\left( 0,\sigma_{h} \right)+N\left( 0,\sigma_{i} \right)$ (7)

In the experiment, motion gain $g$ is varied across trials and observers judge whether $m_{i}$ was to the left or right. The resulting psychometric function describes the probability of judgements in a particular direction (e.g. rightward) as a function of motion gain. Following standard signal detection theory (e.g. Jones, 2016), $m_{i}$ is an internal decision variable. Hence the choice ‘appeared to move rightward’ corresponds to $m_{i}>0$. From signal detection theory we define:

$d=\frac{\mu_{m_{i}}}{\sigma_{m_{i}}}$ (8)

such that the probability of choosing rightward is given by:

$P=\frac{\lambda}{2}+\left( 1-\lambda\right)\Phi\left( \frac{d}{\sqrt{2}} \right)$ (9)

where $\lambda$ is the lapse rate and $\Phi$ is the cumulative distribution function of the standard normal distribution.

By inspection, from Eqn(7):

$\mu_{m_{i}}={(b}_{i}\left( g-1 \right)+1)\mu_{H}$ (10)

Variances sum, so again from Eqn (7):

$\sigma_{m_{i}}=\sqrt{\left( b_{i}\left( g-1 \right)+1 \right)^{2}\sigma_{H}^{2}+\sigma_{h}^{2}+\sigma_{i}^{2}}$ (11)

The point of subjective equality (PSE) occurs when $\mu_{m_{i}}=0$. At this point $b_{i}=1/\left( 1-g \right)$. If there is no bias (i.e. $b_{i}$ = 1), then the PSE occurs when $g$ = 0 i.e. the stimulus is stationary in the speaker/LED ring. This makes sense because no bias means that sensed image motion $i$ for a stationary stimulus is equal and opposite to the sensed head movement $h$ (recall that for our visual stimuli, we provide a head-centred fixation point to inhibit eye rotation in the skull). If $b_{i}$ > 1, then the PSE occurs when $g$ > 1. This describes the head-movement equivalent of a Filehne illusion (Filehne, 1922; Freeman, 2007; Haarmeier & Thier, 1996; Mack & Herman, 1973), in which stationary objects appear to move opposite to a smooth eye pursuit. In order to null the Filehne illusion, the stimulus therefore needs to move in the same direction as the pursuit, equivalent to a positive motion gain. For $b_{i}$ < 1, the opposite is true.


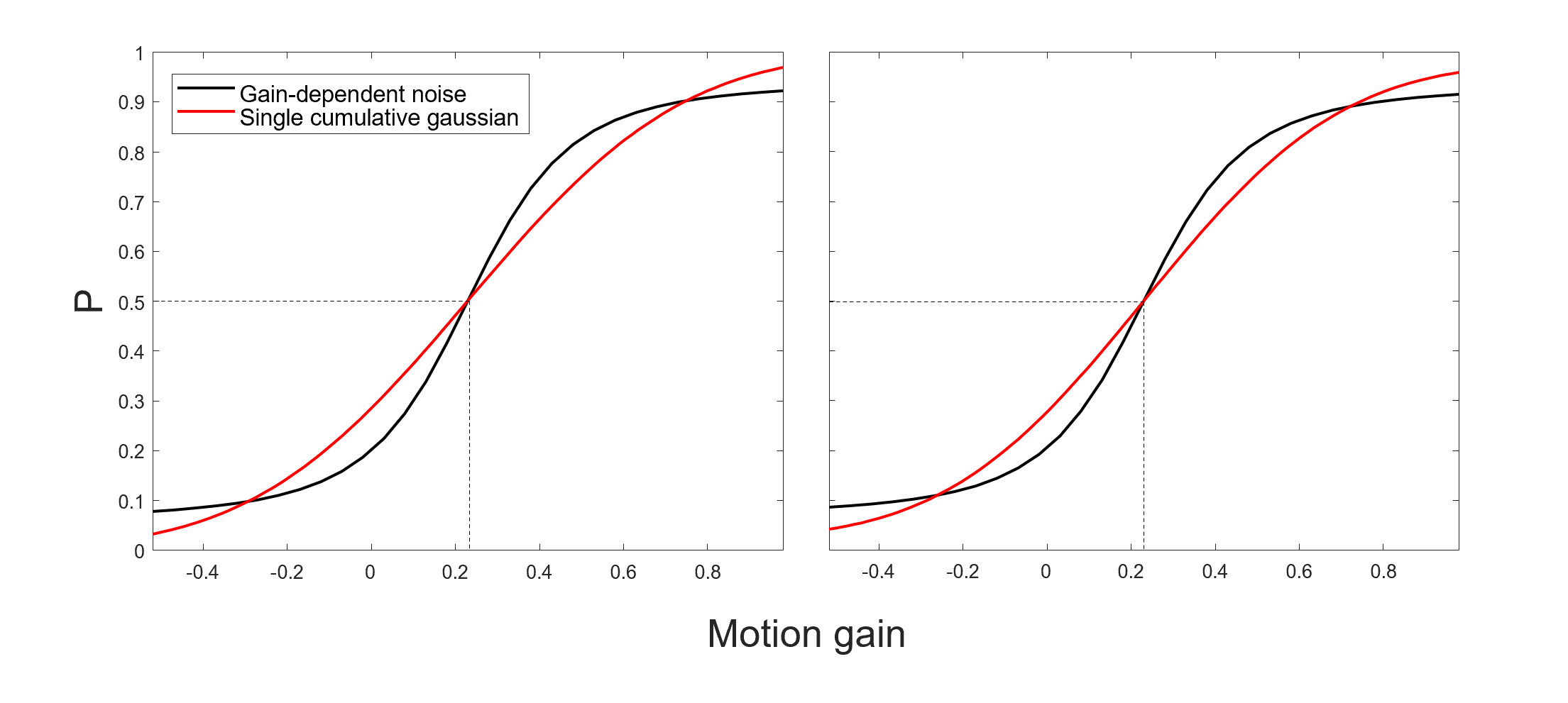


Figure 1: Left: The black curve shows a psychometric function based on gain-dependent noise with $\left\{ \mu_{H}, \sigma_{H}^{2}, \sigma_{h}^{2}, \sigma_{i}^{2}, b_{i},\lambda\right\}=\left\{ 10, 40, 6, 3, 1.3, 0 \right\}$ (note that these parameters do not reflect the typical values used in this experiment, but were deliberately chosen to demonstrate the difference between the standard approach and the present approach). The red curve is the best fitting cumulative Gaussian as determined by the Palamedes toolbox, again with $\lambda=0$. Right: This time with lapse-rate $\lambda=0.02$ for the gain-dependent noise psychometric function, and constrained to vary no larger than 0.02 for the single cumulative Gaussian (Prins, 2012).

If the head movement did not vary across trials ($\sigma_{H}^{2}$ = 0), then the sum $\sigma_{h}^{2}+\sigma_{i}^{2}$ in Eqn (11) could easily be recovered from the best-fitting cumulative Gaussian as per standard fitting of psychometric functions. However, $\sigma_{H}^{2}\neq0$. Variable head movements make the recovery of $\sigma_{h}^{2}+\sigma_{i}^{2}$more complicated because they act as an external source of ‘gain-dependent’ noise that varies with motion gain across the psychometric function. Fitting a single cumulative Gaussian is an approximation at best, as demonstrated in Figure 1. The black curves show example psychometric functions based on the formulae above and parameter values given in the legend; the red curves the best-fitting single cumulative Gaussian. The difference between the two panels is whether lapse rates are included or not. Including gain-dependent noise has two main effects: (1) the asymptotes of the psychometric function move away from $P$= 0 and 1; (2) the slope becomes steeper and is not well fit by a single cumulative Gaussian. The degree to which the gain-dependent noise causes substantial departures from the standard fit depends on the relationship between the values of $\mu_{H}, \sigma_{H}^{2}, \sigma_{h}^{2}, \sigma_{i}^{2}, b_{i}$ and whether lapse-rate is allowed to vary in the standard fit.

***Fitting procedure***

To tackle these issues, we fit psychometric functions to our unimodal condition data based on the formulae above, using the measured head movements to estimate the mean and standard deviation of $H$. The latter were obtained by fitting a Gaussian distribution to the histogram of these movements for the trials making up that psychometric functions. We fixed the variance of the head-movement signal $\sigma_{h}^{2}$in a separate experiment (see Appendix A) because this allowed us to estimate the correlation between cues when evaluating a correlated cue-combination model as discussed below. We note that for the empirical parameter values intrinsic to our observers we did not find much difference between fitting a gain-dependent noise psychometric function and a standard single cumulative Gaussian. One likely explanation for this similarity was that the head movements were relatively consistent ($\sigma_{H}^{2}$ low) given the repetitive nature of the task. But also, we allowed lapse rate to be a (constrained) free parameter when fitting a single cumulative Gaussian ($\lambda\leq0.02)$. As shown in Figure 1B, lapse rate can mimic the asymptotic behaviour of the gain-dependent psychometric function, albeit for the wrong reasons.

**Bimodal conditions**

As explained in the main text, quantitative predictions for bimodal performance start with the idea that cue combination is a weighted average of individual cues, where the weights are the reciprocal of each cue’s precision. When precision is expressed as the variance, the result is a maximum likelihood estimate (see Ernst & Banks, 2002). During self-motion, cue combination is made more complicated because different modalities reside in different coordinate frames: cues must be ‘promoted’ into a common reference frame (Landy et al., 1995). Here we hypothesise that for vision and hearing the common reference frame is body-centred. In the case of our experiment, this means that both modalities use a head rotation signal to interpret image motion, and that combination occurs beyond this ‘compensation’ process. In other words, the perceptual system combines body-centred cues. To investigate, we develop two models, one where body-centred cues are combined using standard MLE principles, and one in which the shared head-rotation signal is taken into account. The first model treats the body-centred cues as independent, whereas the second treats them as partially correlated and follows the logic detailed in Oruç et al. (2003). In both cases, the gain-dependent noise presents an obstacle because, as discussed above, the resulting psychometric functions on which the weights are based are not a single cumulative Gaussian. For this reason, we follow the logic above and define the models at the level of the psychometric function as follows.

***Combining uncorrelated body-centred cues***

From Eqn (11), the variance of the body-centred audio and visual cues is given by:

$\sigma_{i_{bc}}^{2}=\left( b_{i}\left( g-1 \right)+1 \right)^{2}\sigma_{H}^{2}+\sigma_{h}^{2}+\sigma_{i}^{2}$ (12)

for $i$ = $a$ and $v$. Defining reliability as $r_{i_{bc}}=1/{\sigma_{i_{bc}}^{2}}$, then the weights are:

$w_{a_{bc}}={r_{a_{bc}}}/\left( r_{a_{bc}}+r_{v_{bc}} \right)$ (13)

$w_{v_{bc}}={r_{v_{bc}}}/\left( r_{a_{bc}}+r_{v_{bc}} \right)$ (14)

Following the logic of the single-cue condition, we need first to determine the decision variable $d_{{av}_{bc}}={\mu_{{av}_{bc}}}/{\sigma_{{av}_{bc}}}$ in order to construct the psychometric function. This time, however, the decision variable will be based on the sum of the weighted body-centred distributions for audio and visual cues:

$d_{{av}_{bc}}=w_{a_{bc}}N\left( \mu_{a_{bc}},\sigma_{a_{bc}} \right)+w_{v_{bc}}N\left( \mu_{v_{bc}},\sigma_{v_{bc}} \right)$ (15)

Given that Eqn (10) defines the mean for a single cue, and Eqn (12) defines its variance, then:

$\mu_{{av}_{bc}}={\mu_{H} \text{. }[w}_{a_{bc}}{(b}_{a}\left( g-1 \right)+1)+w_{v_{bc}}{(b}_{v}\left( g-1 \right)+1)]$ (16)

$\sigma_{{av}_{bc}}= \sqrt{w_{a_{bc}}^{2}\sigma_{a_{bc}}^{2}+w_{v_{bc}}^{2}\sigma_{v_{bc}}^{2}}$ (17)

The psychometric function is then derived using Eqn (9).

***Combining correlated body-centred cues***

Following Oruç et al. (2003), the reliabilities of correlated cues must be corrected for the shared noise. Specifically:

$r_{a_{bc}}^{'}=r_{a_{bc}}-\rho\sqrt{r_{a_{bc}}+r_{v_{bc}}}$ (18)

$r_{v_{bc}}^{'}=r_{v_{bc}}-\rho\sqrt{r_{a_{bc}}+r_{v_{bc}}}$ (19)

These define a new wet of weights $w_{a_{bc}}^{'}$ and $w_{v_{bc}}^{'}$ using the same logic as Eqns (13) and (14). The decision variable $d_{{av}_{bc}}$ then has mean and standard deviation:

$\mu_{{av}_{bc}}^{'}=\mu_{H} . [w_{a_{bc}}^{'}{(b}_{a}\left( g-1 \right)+1)+w_{v_{bc}}^{'}{(b}_{v}\left( g-1 \right)+1)]$ (20)

$\sigma_{{av}_{bc}}^{'}= \sqrt{w_{a_{bc}}^{'2}\sigma_{a_{bc}}^{2}+w_{v_{bc}}^{'2}\sigma_{v_{bc}}^{2}-\frac{2\rho w_{a_{bc}}^{'}w_{v_{bc}}^{'}}{\sqrt{r_{a_{bc}}+r_{v_{bc}}}}}$ (21)

where Eqn (21) is based on Eqn (6) of Oruc et al.

***Fitting procedure and model evaluation***

The parameters defining the psychometric functions for the correlated and uncorrelated cue combination models are all fixed by the unimodal single-cue conditions, apart from the correlation $\rho$ and the ubiquitous lapse rate $\lambda$. The correlation could be made free to vary when fitting the correlated cue combination model to the bimodal data. However, we have recently developed a technique to measure the variance of the head movement signal $\sigma_{h}^{2}$, which therefore allows us to fix the correlation as described below. Models are then evaluated by comparing goodness-of fit measures at the level of the psychometric functions, as opposed to the normal route which is based on PSEs and precision measures (e.g. thresholds, slopes). To reiterate, the reason this standard procedure could fail is because the use of a single cumulative Gaussian with an accompanying lapse rate is at best an approximation.

To obtain the correlation, recall from Eqn (7) that the body-centred audio and visual cues ($m_{a}$ and $m_{v}$) share exact copies of the head-movement signal ($h$) but scaled copies of actual head movements ($H$). The scale is determined by their biases and the motion gain. Noting that the correlation between $m_{a}$ and $m_{v}$ is their covariance divided the square-root of the product of their variances, it can be shown that:

$\rho=\frac{\left( k_{a}k_{v}\sigma_{H}^{2}+\sigma_{h}^{2} \right)}{\sqrt{\left( \sigma_{a}^{2}+k_{a}^{2}\sigma_{H}^{2}+\sigma_{h}^{2} \right)\left( \sigma_{v}^{2}+k_{v}^{2}\sigma_{H}^{2}+\sigma_{h}^{2} \right)}}$ (22)

where ${k_{i}=b}_{i}\left( g-1 \right)+1$ for $i$ = $a$ and $v$.

**References**

Aubert, H. (1886). Die Bewegungsempfindung. *Pflüger, Archiv für die Gesammte Physiologie des Menschen und der Thiere*, *39*(1), 347–370. https://doi.org/10.1007/BF01612166

Dichgans, J., Wist, E., Diener, H. C., & Brandt, Th. (1975). The Aubert-Fleischl phenomenon: A temporal frequency effect on perceived velocity in afferent motion perception. *Experimental Brain Research*, *23*(5). https://doi.org/10.1007/BF00234920

Ernst, M. O., & Banks, M. S. (2002). Humans integrate visual and haptic information in a statistically optimal fashion. *Nature*, *415*(6870), 429–433. https://doi.org/10.1038/415429a

Filehne, W. (1922). Über das optische Wahrnehmen von Bewegungen. *Zeitschrift Für Sinnesphysiology*, *53*, 134.

Freeman, T. C. A. (2001). Transducer models of head-centred motion perception. *Vision Research*, *41*(21), 2741–2755. https://doi.org/10.1016/S0042-6989(01)00159-6

Freeman, T. C. A. (2007). Simultaneous adaptation of retinal and extra-retinal motion signals. *Vision Research*, *47*(27), 3373–3384. https://doi.org/10.1016/j.visres.2007.10.002

Freeman, T. C. A., & Banks, M. S. (1998). Perceived head-centric speed is affected by both extra-retinal and retinal errors. *Vision Research*, *38*(7), 941–945. https://doi.org/10.1016/S0042-6989(97)00395-7

Garzorz, I. T., Freeman, T. C. A., Ernst, M. O., & MacNeilage, P. R. (2018). Insufficient compensation for self-motion during perception of object speed: The vestibular Aubert-Fleischl phenomenon. *Journal of Vision*, *18*(13), 9. https://doi.org/10.1167/18.13.9

Haarmeier, T., & Thier, P. (1996). Modification of the filehne illusion by conditioning visual stimuli. *Vision Research*, *36*(5), 741–750. https://doi.org/10.1016/0042-6989(95)00154-9

Haynes, J. D., Gallagher, M., Culling, J. F., & Freeman, T. C. A. (2024). The precision of signals encoding active self-movement. *Journal of Neurophysiology*. https://doi.org/10.1152/jn.00370.2023

Jones, P. R. (2016). A tutorial on cue combination and Signal Detection Theory: Using changes in sensitivity to evaluate how observers integrate sensory information. *Journal of Mathematical Psychology*, *73*, 117–139. https://doi.org/10.1016/j.jmp.2016.04.006

Landy, M. S., Maloney, L. T., Johnston, E. B., & Young, M. (1995). Measurement and modeling of depth cue combination: In defense of weak fusion. *Vision Research*, *35*(3), 389–412. https://doi.org/10.1016/0042-6989(94)00176-M

Mack, A., & Herman, E. (1973). Position Constancy during Pursuit Eye Movement: An Investigation of the Filehne Illusion. *Quarterly Journal of Experimental Psychology*, *25*(1), 71–84. https://doi.org/10.1080/14640747308400324

Oruç, İ., Maloney, L. T., & Landy, M. S. (2003). Weighted linear cue combination with possibly correlated error. *Vision Research*, *43*(23), 2451–2468. https://doi.org/10.1016/S0042-6989(03)00435-8

Prins, N. (2012). The psychometric function: The lapse rate revisited. *Journal of Vision*, *12*(6), 25. https://doi.org/10.1167/12.6.25
